# Vascular Epiphytic Medicinal Plants as Sources of Therapeutic Agents: Their Ethnopharmacological Uses, Chemical Composition, and Biological Activities

**DOI:** 10.3390/biom10020181

**Published:** 2020-01-24

**Authors:** Ari Satia Nugraha, Bawon Triatmoko, Phurpa Wangchuk, Paul A. Keller

**Affiliations:** 1 Drug Utilisation and Discovery Research Group, Faculty of Pharmacy, University of Jember, Jember, Jawa Timur 68121, Indonesia; bawon.farmasi@unej.ac.id; 2 Centre for Biodiscovery and Molecular Development of Therapeutics, Australian Institute of Tropical Health and Medicine, James Cook University, Cairns, QLD 4878, Australia; phurpa.wangchuk@jcu.edu.au; 3 School of Chemistry and Molecular Bioscience and Molecular Horizons, University of Wollongong, and Illawarra Health & Medical Research Institute, Wollongong, NSW 2522 Australia

**Keywords:** epiphytes, medicinal plants, phytochemistry, pharmacology, drug leads

## Abstract

This is an extensive review on epiphytic plants that have been used traditionally as medicines. It provides information on 185 epiphytes and their traditional medicinal uses, regions where Indigenous people use the plants, parts of the plants used as medicines and their preparation, and their reported phytochemical properties and pharmacological properties aligned with their traditional uses. These epiphytic medicinal plants are able to produce a range of secondary metabolites, including alkaloids, and a total of 842 phytochemicals have been identified to date. As many as 71 epiphytic medicinal plants were studied for their biological activities, showing promising pharmacological activities, including as anti-inflammatory, antimicrobial, and anticancer agents. There are several species that were not investigated for their activities and are worthy of exploration. These epipythes have the potential to furnish drug lead compounds, especially for treating cancers, and thus warrant indepth investigations.

## 1. Introduction

Epiphytes are plants that grow on other plants and are often known as air plants. They are mostly found in moist tropical areas on canopy tree-tops, where they exploit the nutrients available from leaf and other organic debris. These plants exist within the plantae and fungi kingdom. The term epiphyte itself was first introduced in 1815 by Charles-François Brisseau de Mirbel in “Eléments de physiologie végétale et de botanique” [1]. Epiphytes can be categorized into vascular and non-vascular epiphytic plants; the latter includes the marchantiophyta (liverworts), anthocerotophyta (hornworts), and bryophyta (mosses). The common epiphytes are mosses, ferns, liverworts, lichens, and the orchids. Epiphytes fall under two major categories: As holo- and hemi-epiphytes. While orchids are a good example of holo-epiphytes, the strangler fig is a hemi-epiphyte. Although geological studies have proposed the existence of epiphytes since the pleistone epoch, an epiphyte was first depicted in “the Badianus Manuscript” by Martinus de la Cruz in 1552, which showed the *Vanilla fragrans*, a hemi-epiphytic orchid, being used by the tribal communities in latin America for fragrance and aroma, usually hung around their neck [1].

Epiphytes have been a source of food and medicine for thousands of years. Since they grow in a unique ecological environment, they produce interesting secondary metabolites that often show exciting biological activities. There are notable reviews on non-vascular epiphytes, bryophyta, regarding their phytochemical and pharmacological activities [2,3,4,5]. There are also extensive reviews on epiphytic lichens covering secondary metabolites and their pharmacological activities [6,7,8,9]. The only available review on vascular epiphytes related to medicinal uses was focused on Orchidaceae [10]. Therefore, to the best of our knowledge, there is no extensive database of vascular epiphytes regarding their medicinal contribution. 

**Figure 1:**
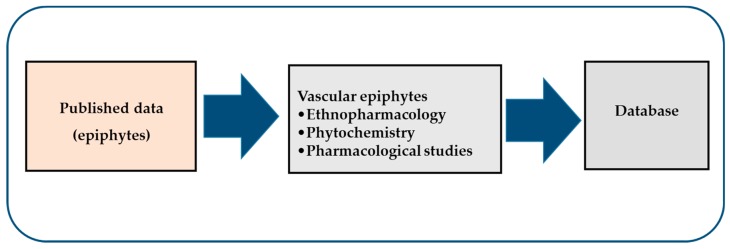
Schematic data collection approach.

There are 27,614 recorded species of vascular epiphytes belonging to 73 families and 913 genera [11]. Vascular epiphyte species are commonly found in pteridophyta, gymnosperms, and angiosperms plant groups, which are mostly found in the moist tropical areas on canopy tree tops, where they exploits the nutrients available from leaf and other organic debris [12,13]. In this study, information on vascular epiphytic medicinal plant species was collected using search engines (Web of Science, Scifinder Scholar, prosea, prota, Google scholar), medicinal plant books (Plant Resources of South-East Asia: Medicinal and Poisonous Plants [14,15,16], Plant Resources of South-East Asia: Cryptogams: Ferns and Fern Allies [17], Mangrove Guide for South-East Asia [18], Medicinal Plants of the Asia-Pacific [19], Medicinal Plants of the Guiana [20], Indian Medicinal Plants [21,22], Medicinal Plants of Bhutan [23], Medicinal and aromatic plants of Indian Ocean islands: Madagascar, Comoros, Seychelles and Mascarenes [24]), and the Indonesian Medicinal Plants Database [25]. Scientific names of the epiphytic medicinal plant species were compared against the Plantlist database for accepted names to avoid redundancy [26]. The time-frame threshold for data coverage was from the earliest available data until early 2020. Nevertheless, empirical knowledge regarding traditional medicinal plants was passed through generations using verbal or written communication, with verbal communication highly practiced by remote tribes [27,28]. It is possible that some oral traditional medical knowledge may not be reported and therefore not captured in this review. In this current study, we collected and reviewed 185 epiphytic medicinal plants reported in the literature, covering ethnomedicinal uses of epiphytes, their phytochemical studies and the pharmacological activities. The data collection approach used is presented in Figure 1.

## 2. Ethnopharmacological Information of Vascular Epiphytic Medicinal Plants 

### 2.1. Vascular Epiphytic Medicinal Plant Species Distribution within Plant Families

**Figure 2:**
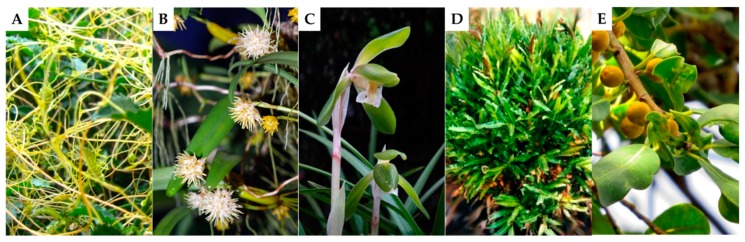
Five most popular medicinal epiphytes. (**A**) *C. filiformis* L. (**B**) *B. odoratissimum* (Sm.) Lindl. ex Wall. (**C**) *C. goeringii* (Rchb.f.) Rchb.f. (**D**) *A. aureum* Limme. (**E**) *F. natalensis* Hochst.

In this component of the study, we collated and analysed 185 of the medicinally used epiphytic plants species using ethnopharmacological information. This data (Table 1) includes the name of species, plant family, areas where the epiphytes are used in traditional medicines, part(s) of the plant being used in medication, how the medicine was prepared, and indications. Of the 185 medicinally used epiphytes, 53 species were ferns (mostly polipodiaceae), with 132 species belonging to the non-fern category. The Orchidaceae family contains the *Dendrobium* genus that contains the highest number of medicinal epiphytes, including 64 orchid species and 20 Dendrobium species. The Orchidaceae epiphytes were the majority of non-fern epiphytes. *Cassytha filiformis* L, *Bulbophyllum odoratissimum* (Sm.) Lindl. ex Wall., *Cymbidium goeringii* Rchb.f.) Rchb.f., *Acrostichum aureum* Limme, and *Ficus natalensis* Hochst. were the five most popular vascular epiphytic medicinal pants used (Figure 2).

### 2.2. Distribution of Vascular Epiphytic Medicinal Plant Species by Country

**Figure 3:**
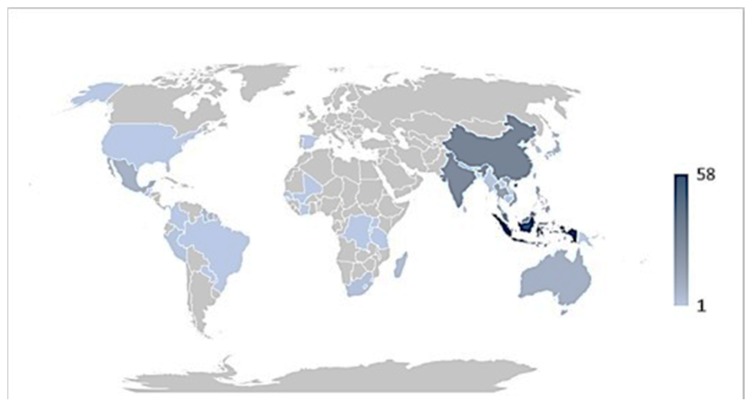
Density map showing a number of epiphytic medicinal plant species used by different countries. The number of species used is proportional to colour intensity.

Based on the available records, the data curation and analysis revealed that the Indigenous Indonesians have used 58 diverse epiphytic medicinal plant species throughout the archipelago and have the highest record compared to other tropical countries (Figure 3). China is second and is well known for its traditional medicine, including the use of epiphytes in medicament preparation. This is followed by the Indigenous Indians, with the well-established Ayurveda as a formal record of Indian medicinal plants. The traditional medicinal plant knowledge of Indonesa has been heavily influenced by Indian culture and enriched by Chinese and Arabian traders since the kingdom era [27].

### 2.3. Parts of Vascular Epiphytic Medicinal Plant Species Used in Traditional Medicines

**Figure 4:**
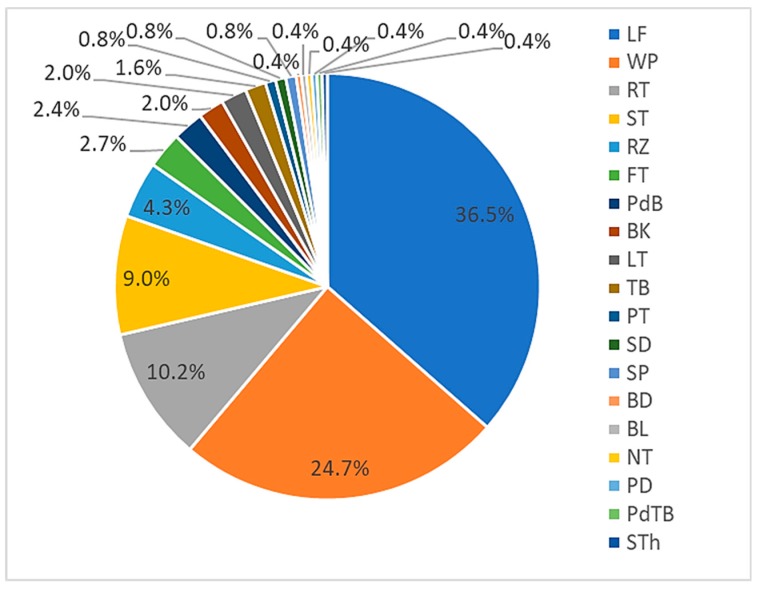
Components of epiphytic plants used in medicinal preparations (represented in percentages). LF: leaf; WP: whole; RT: root; ST: stem, RZ: rhizome; FT: fruit; PdB: pseudobulbs; BK: bark; LT: latex; TB: tuber; PT: pith; SD: seed; SP: spore; BD: buds; BL: bulbs: NT: nutmeg; PD: pedi; PdTB: pseudotuber; STh: sheath.

This review determined that leaves were the main plant components used in the traditional medicines (Figure 4). This was expected given they are more easily harvested (without excessive tools) and processed compared to other plant parts, e.g., the root and stem. As some epiphytes have a small biomass compared to higher trees, the whole plant is commonly harvested in medicament preparation. Interestingly, almost half of epiphytic medicinal plants were ferns, in which the stem-like stipe is prepared for medicine. Without haustoria (a specialised absorbing structure of a parasitic plant), the root and rhizome of epiphytic medicinal plants are easily harvested and prepared.

### 2.4. Modes of Preparation and Dosage of Administration of Vascular Epiphytic Medicinal Plant Species in Traditional Medicines

**Figure 5:**
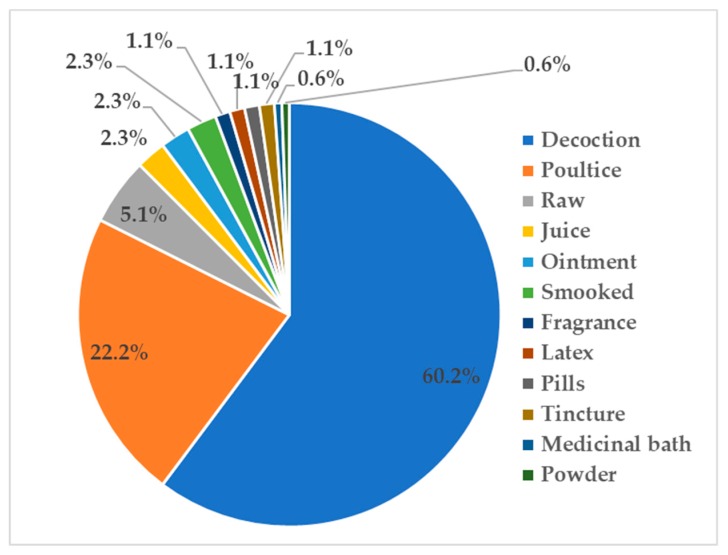
Modes of preparation and administration of epiphytic medicinal plants (represented in percentages).

Generally, medicinally active secondary metabolites have a water solubility problem likely related to the lipophilic moieties in their structures [29]. Using boiling water, decoctions are able to increase the yield of secondary metabolites extracted from medicinal plants. Therefore, it is not surprising that decoctions are commonly used in traditional medicine preparations from plants (Figure 5). External applications are also commonly practiced in traditional medicinal therapies, including poultice (moist mass of material), raw, or less processed medicine. Poultices were commonly prepared for skin diseases while a decoction was ingested for internal infectious diseases (i.e., fever).

### 2.5. Category of Diseases Treated by Vascular Epiphytic Medicinal Plant Species

**Figure 6:**
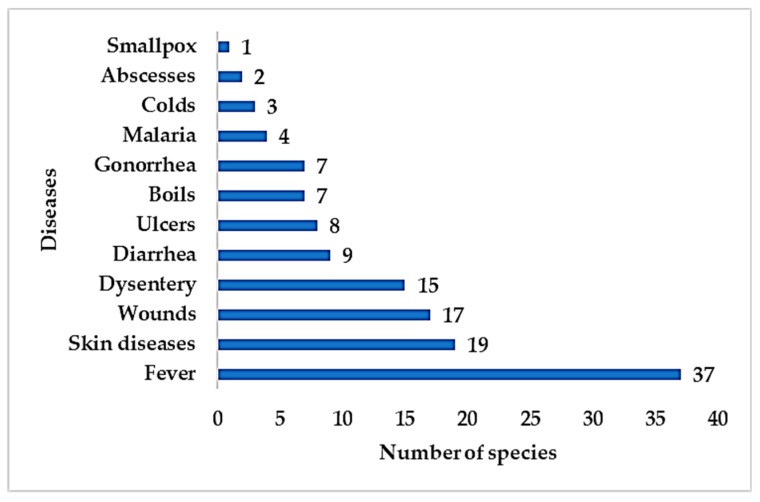
Number of epiphytic medicinal plant species used traditionally to treat infectious diseases.

Interestingly, epiphytes have been used for treating various ailments, including both infectious and non-infectious diseases. Traditional communities described infectious diseases related to skin diseases (wounds, boils, ulcers, abscesses, smallpox) and non-skin diseases (fever, diarrhoea, ulcers, colds, worm infections, and malaria). A total of 54 epiphytic medicinal plant species were prescribed to treat skin diseases while 81 species to treat non-skin infectious diseases (Figure 6).

Hygiene has been a serious issue in traditional communities as it gives rise to infectious diseases. Fever is a common symptom of pathogenic infection and has been treated using medicinal plants, including epiphytes. Hygiene issues are also a common cause of skin disease, wounds, dysentery, and diarrhoea in traditional communities.

## 3. Phytochemical Composition of Vascular Epiphytic Medicinal Plants

Epiphytes belong to a distinctive plant class as they do not survive in soil and this influences the secondary metabolites present. Epiphytes are physically removed from the terrestrial soil nutrient pool and grow upon other plants in canopy habitats, shaping epiphyte morphologies by the method in which they acquire nutrients [30]. Nutrients, such as nitrogen and phosphorus, are obtained from different sources, including canopy debris (through fall) and host tree foliar leaching [30], the latter influencing canopy soil nutrient cycling [31,32]. In the conversion of sunlight into chemical energy, the epiphyte often uses a specific carbon fixation pathway (CAM: Crassulacean acid metabolism) as a result of harsh environmental conditions [33], making them unique and thus worthwhile for scientific studies.

**Figure 7:**
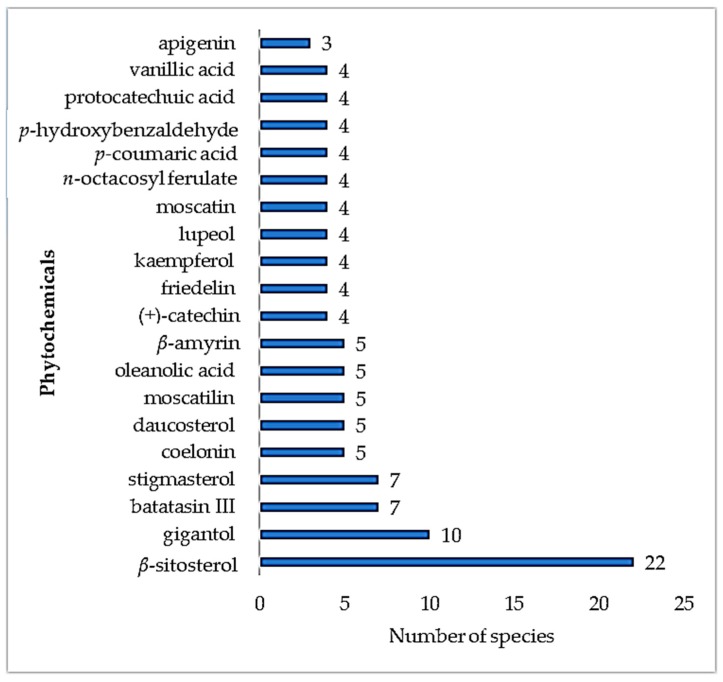
Number of epiphytic medicinal plant species producing the same secondary metabolites.

In the early 20th century, laboratory-based research on epiphytes studied the plant’s production of alkaloids, cyanogenetic, and organic sulfur compounds, with the plants producing limited quantities of these compounds [34]. Common plant steroids, e.g., β-sitosterol, have been shown to be present in 22 different epiphytic medicinal plants (Figure 7). This is possibly due to the function of the steroids as structural cell wall components, giving rise to a wide distribution across plant families and species. A further example of a common plant steroid present is stigmasterol.

Table 2 lists the secondary metabolites identified in epiphytic medicinal plants and details the species, isolated compounds, and provides references. Currently, only 69 species have been phytochemically studied (23 fern and 46 non-fern epiphytes) and 842 molecules have been isolated from these epiphytic plants. Analysis of the literature showed epiphytes were able to produce a range of secondary metabolites, including terpenes and flavonoids, with no alkaloids being isolated from epiphytic fern medicinal plants thus far. *β*-Sitosterol, a common phytosterol in higher plants, was reported across fern genera. Interestingly, there is one unique terpene produced, hopane, which is commonly called fern sterol. Common flavonoids, such as kaempferol, quercetin, and flavan-3-ol derivatives (catechin), were also reported across the epiphytic ferns. Epiphytic pteridaceae, *Acrostichum aureum* Limme, is rich in quercetin [35]. Further analysis showed there were more secondary metabolites reported from non-fern epiphytic medicinal plants than from fern epiphytic medicinal plants, including terpene derivatives, flavonoids, and alkaloids. Included were flavanone, flavone, and flavonol derivatives but no flavan-3-ols were reported in these epiphytes so far. In the non-fern epiphytes, there were more phytochemical studies on orchid genera with additional classes of compounds reported, including penantrene derivatives (flavanthrinin, nudol, fimbriol B) [36,37] from the *Bulbophyllum* genus and the alkaloid dendrobine from the *Dendrobium* genus [38].

Therefore, while epiphytes may have limitations in accessing nutrients, adaptation has enabled them to successfully survive these environments. Studies on numerous medicinal epiphytes show that the unique environment does not constrain the plants from producing different types of secondary metabolites. These include terpenes, flavonoids, and alkaloids, especially the non-fern epiphytic medicinal plants.

## 4. Pharmacological Activities of Vascular Epiphytic Medicinal Plants

The pharmacological activities of medicinal epiphytes are summarised in Table 1, including the plant species, ethnopharmacological indication, and pharmacological test results. The ethnopharmacological uses of each plant are also present for a correlation and comparison with the pharmacological activities. There are a large number of phytochemical studies on the four fern-epiphytes (*Stenochlaena palustris* (Burm. F.) Bedd., *Botrychum lanuginosum* Wall.ex Hook & Grev., *Pyrrosia petiolosa* (Christ) Ching, *Psilotum nudum* (L.) P. Beauv) without any biological activity testing reported. This occurred to four non-fern epiphytes (*Bulbophyllum vaginatum* (Lindl.) Rchb.f, *Mycaranthes pannea* (Lindl.) S.C.Chen & J.J.Wood, *Pholidota articulata* Lindl., *Viscum ovalifolium* DC) and non-fern epiphytic medicinal plants. This lack of pharmacological testing limits scientific support for the traditional uses of these plants.

From the 191 collected records of epiphytic medicinal plants, around 71 species were subjected to bioactivity testing, with 25 of these species using crude extract samples. Although this testing represents almost 50% of the species examined, only a few of the pharmacological tests were related to ethnopharmacological claims. Here, we discuss selected species where the outcomes indicated a coherent relationship between bioactivities and traditional claims.

### 4.1. Infectious Disease Therapy

Research on epiphytes that have been used in infectious disease therapy include in wound healing, dysentery, and skin infections. A study on the methanol extract of *Adiantum caudatum* L., Mant showed anti-fungal activity against common fungi found in wounds (*Aspergilus* and *Candida* species) [39], including *Aspergillus flavus*, *A*. *spinulosus*, *A. nidulans*, and *Candida albicans*, with minimum inhibitory concentration (MIC) values of 15.6, 15.6, 31.2, and 3.9 µg/mL, respectively. Gallic acid was one of the bioactive constituents [40]. The methanol extract of *Ficus natalensis* Hochst (a semi-epiphytic plant) showed anti-malarial activity against *Plasmodium falciparum*, with an half maximal inhibitory concentration (IC_50_) value of 41.7 µg/mL, and weak bactericidal activity against *Staphylococcus aureus*, with an MIC value of 99 µg/mL [41]. These results became preliminary data for confirming its traditional uses as malarial fever therapy and wound healing. Phytochemical studies on *Pyrrosia sheareri* (Bak.) Ching successfully isolated several compounds and were subjected to anti-oxidant testing. While this was not in line with the plant’s ethnomedical uses for dysentery therapy [42], one of the isolated constituents was protocateuchic acid, which is known to possess anti-bacterial activity. It implies that the traditional uses of the epiphyte was for bacillary dysentery therapy.

### 4.2. Non-Infectious/Degenerative Disease-Related Therapy

An exploration on *Drynaria* species, highly prescribed in bone fracture therapy, successfully isolated flavonoid constituents that induce osteoblast proliferation [43]. Previous studies on *Acrostichum aureum* Limme failed to show its anti-bacterial activities [44] contrary to its traditional claims in wound management. However, patriscabratine **257** was isolated from the defatted methanol extract of whole plant of *A. aureum*, and subsequent testing showed it possessed anti-cancer activity in gastric cells and this supprted the traditional use of the plant in peptic ulcer therapy [35]. A decoction from the epiphyte *Ficus deltoida* has been used to treat diabetes. A study on the hot aqueous extract of this plant revealed anti-hyperglycemic activity by stimulating insulin secretion up to seven-fold. Furthermore, its activity mechanism was related to both the K^+^_ATP_-dependant and -non-dependant insulin secretion pathway [45]. However, further studies are required to identify the constituents responsible for the anti-hyperglycaemic activity.

The Indigenous people of Paraguay have used *Catasetum barbatum* Lindley to topically treat inflammation. Four bioactive compounds were isolated from this species and 2,7-dihydroxy-3,4,8-trimethoxyphenanthrene (confusarin) **595** showed the highest anti-inflammatory activity [46]. The study also revealed the compound to be a non-competitive inhibitor of the H_1_-receptor.

From the polypodiaceae family, the rhizome of *Phymatodes scolopendria* (burm.) Ching has been used to treat respiratory disorders. A bioassay-guided phytochemical study on *Phymatodes scolopendria* (Burm. f.) Pic. Serm. isolated 1,2-benzopyrone (coumarin) **209** as a bronchodilator [47].

## 5. Epiphytic Plant–Host Interactions on Secondary Metabolite Tapping

Secondary metabolite tapping has been an interesting study to reveal the molecular interactions between epiphytes and their host. This interaction was more visible when a physical channel between the two were developed. This channel (haustorium) made an epiphytic plant act as a parasite that enabled the plant to harvest molecular components from the host plant. A study on *Scurulla oortiana* (Korth.) Danser growth in three different host species (*Citrus maxima*, *Persea Americana*, and *Camellia sinensis*) identified three secondary metabolites (quercitrin, isoquercitrin, and rutin) in the *S. oortiana* (Korth.) Danser epiphyte growing on the three hosts [48]. Interestingly, extensive chromatographic and spectroscopic studies discovered that the flavonoids found in the *S. oortiana* (Korth.) Danser were independent of the host plants [48]. Secondary metabolite production in a host plant can also be triggered by the existence of a parasite, as discussed in a study on *Tapirira guianensis* infested by *Phoradendron perrottetii*, in which infested branches produced more tannin compare to non-infested branches, with infestation inducing a systemic response [48].

## 6. Conclusions

Epiphytes are the most beautiful vascular plants and contain interesting phytochemicals and possess exciting pharmacological activities. An analysis of the literature revealed 185 epiphytes that are used in traditional medicine, in which phytochemical studies identified a total of 842 secondary metabolites. Only 71 epiphytic medicinal plants were studied for their pharmacological activities and showed promising pharmacological activities, including anti-inflammatory, antimicrobial, and anticancer. Several species were not investigated for their activities and are worthy of exploration, including epiphytes from the Araceae (*P. fragantissimum*), Aralliaceae (*S. caudata, S. elliptica, S. elliptifoliola, S. oxyphylla, S. simulans*), and Asclepidaceae (*Asclopidae sp., D. acuminate, D. benghalensis, D. imbricate, D. major, D. nummularia, D. platyphylla, D. purpurea, Toxocarpus sp*) families, in which no phytochemical and pharmacological studies had been reported. These species have been used by Indigenous populations to treat both degenerative and nondegenerative diseases. It is known that there are examples of Indigenous populations living in protected forest reserves (e.g., in Indonesia) where epiphytes are used in their medicine, e.g., some species of *Dischidia* are used to treat fever, eczema, herpes etc.; these plants have not yet been studied. Therefore, the possibility of responsible bioprospecting exists (in compliance with the Nagoya protocol), which would be invaluable in biodiscovery knowledge as well as in mutual benefit sharing agreements.

## Figures and Tables

**Table 1:** Ethnopharmacological database of epiphytic medicinal plants.

No	Epiphyte Species	Location	Part of Plants	Preparation and Route of Administration	Indication (traditional)	Pharmacological Testing (modern)
**Fern species**						
	**Adiantaceae**					
1	*Adiantum caudatum* L.	India, Indonesia, Malaysia	LF	Decoction	Cough, heal wound, cold, tumors of spleen, liver and other viscera, skin diseases, bronchitis, and inflammatory diseases [40,49,50]	Antimicrobial (MeOH extract, gram +, -, fungi) [40]
	**Aspleanceae**					
2	*Asplenium nidus* L.	Tahiti, Malaysia, Philippines, Vanuatu, Indonesia	LF, WP	Ointment, decoction, eaten	Headache, hair loss (pounded leaves mixed with coconut oil), ease labor, fever (decoction), contraceptive, depurative, sedative agents. edible food (young leaves), ornament, anti-inflammation, promote blood circulation [51,52,53]	Antioxidative (MeOH extract, DPPH), tyrosinase inhibiting (MeOH extract, microtitre), antibacterial (MeOH extract) [44]
3	*Asplenium macrophyllum* Sw.	India	LF	Decoction	As laxative, emetic, diuretic, anthelmintic agent, to treat ophthalmia, jaundice, spleen diseases [52,54]	
4	*Asplenium polydon* G. Foster var *bipinnatum* (Sledge)	India	LF	Decoction, paste	Promote labor, tumor [55]	
5	*Asplenium serratum* L.	Columbia, Peru	na	Not mentioned	Liver problem, stomachache, ovary inflammation [52,56]	
	**Blechnaceae**					
6	*Stenochlaena palustris* (Burm. F.) Bedd.	Indonesia, India	LF, RZ	Eaten, decoction, poultice	Young reddish leaves are used as food, leaves are used to treat fever, skin diseases, throat, and gastric ulcer, as antibacterial, rhizome and leaves are used to treat burns and ulcers, as cooling agent [18,57]	
	**Davalliaceae**					
7	*Davallia denticulata* (Burm. f.) Mett. ex Kuhn	Malaysia, Indonesia	RT	Decoction	Gout, pain, as tonic [49,58]	
8	*Araiostegia divaricata* (Blume) M. Kato	China, Taiwan	WP	Not mentioned	Joint pain [59]	Anti-psoriasis [60], antioxidant (water extract, DPPH) [61]
9	*Davallia parvula* Wall. Ex Hook. & Grev.		na	Not mentioned	Not mentioned [18,62]	
10	*Davallia solida* (G. Forst.) Sw.	Tahiti, Fiji, other Polynesian	WP	Decoction (external and internal)	Dysmennorrhea, luochorea, uterine hemorrhage, sore throat, asthma, constipation, fracture, fish sting, promote health pregnancy, as a bath for newborn, anti-microbial [53,63,64,65]	Antioxidant (extract, ABTS) [61], antioxidant (DPPH, all isolates) [66], anti-neurotoxicity (extract, (Neuro-2a cells, ATCC CCL-131) [67], C-terminal cytosolic domain of P-pg [68], anti-skin aging [69]
11	*Leucostegia immersa* Wall. ex C. Presl	Nepal	RZ	Decoction, paste	Boils (paste), constipation (decoction), as antibacterial (paste) [70]	
	**Gesneriaceae**					
12	*Aeschynanthus radicans* Jack	Malaysia	LF	Decoction	Headache [19]	
13	*Cyrtandra sp*	Indonesia	LF	Poultice	Skin ailments [71]	
	**Hymenophyllaceae**					
14	*Hymenophyllum polyanthos* Sw.	Suriname	WP	Burnt (smoke inhaling), decoction	Dizziness (insanity), pain, cramps [72]	
15	*Hymenophyllum javanicum* Spreng.	India	WP	Smoke together with garlic and onions	Headache [55]	
	**Lycopodiaceae**					
16	*Huperzia carinata* (Desv. ex Poir.) Trevis	South-East Asia	WP	Ointment	Stimulate hair growth [73]	Anti-acetylcholinesterase (**74**, **75**, **76**, colorimetric Ellman method) [74]
17	*Huperzia phlegmaria* (L.) Rothm	South-East Asia, India	WP	Ointment	Stimulate hair growth, skin diseases [75,76]	Cytotoxic activities against HuCCA-1, A-549, HepG2, and MOLT-3 cancer cell lines (**81**, **79**, **77**) [77]
18	*Huperzia megastachya* (Baker) Tardieu	Madagascar	LF	Decoction (infusion)	Tonic [78]	
19	*Huperzia obtusifolia* (Sw.) Rothm.	Madagascar	LF	Decoction (infusion)	Tonic [78]	
	**Nephrolepidaceae**					
20	*Nephrolepis acutifolia* (Desv.) Christ	Malaysia	WP	Boiled, eaten	Food [79]	
21	*Nephrolepis biserrata* (Sw.) Schott	Malaysia, Indonesia, Ivory Coast, New Guinea	LF, RZ, WP	Decoction, cooked	Leaves are used to treat boils, blister, abscesses, sores, and cough. Rhizomes are used as edible food [80,81]	Antibacterial (extract) [82]
	**Oleandraceae**					
22	*Nephrolepis cordifolia* (L.) C. Presl	India	RZ	Decoction (fresh leaves)	Cough, rheumatism, chest congestion, nose blockage, loss appetites, infection (antibacterial), pinnae is used to treat cough, wounds, jaundice, anti-fungal, styptic, anti-tussive [57]	Antibacterial, anti-fungal (extract fractions aerial part) [83]
23	*Oleandra musifolia* (Blume) C. Presl	Philippines, India	ST	Decoction	Anthelmintic, emmenagogue, antidote (snake bite) [70,84]	
	**Opioglossaceae**					
24	*Botrychum lanuginosum* Wall.ex Hook & Grev.	India	WP	Decoction, paste	Antibacterial, anti-dysentery agents [57]	
25	*Ophioglossum pendulum* L.	Indonesia, Philippines	LF	Ointment, decoction.	Hair treatment (crushed leaves), cough (decocotion), rid the first feces (spores), ornament [85]	Cell activator, skin whitening agent and antioxidant (patent, mixed with other *Ophioglossum* species) [86], anti-diarrhea (stipe MeOH extract, rabit jejenum) [86]
	**Polypodiaceae**					
26	*Pyrrosia piloselloides* (L.) M.G. Price	Indonesia, Malaysia, China, Philippines, Pacific islands	LF	Decoction (internal), chewed, poultice (external)	Smallpox, rashes, gonorrhea, dysentery, tuberculosis, urinary tract infection, headache, cough, gum inflammation, tooth sockets, eczema, coagulate blood [87,88,89,90]	Antibacterial, anti-fungal (extracts) [91]
27	*Drynaria rigidula* (Sw.) Bedd.	Indonesia, Philippines, Treasury Island	LF, RZ	Decoction, chewing	Gonorrhea, dysentery (rhizome, decoction), and seasickness (chewed) [21]	*n*-Hexane, dichloromethane and ethyl acetate fractions from both rhizome and leaves of *Drynaria rigidula* were screened for activity against *Plasmodium falciparum*, *Mycobacterium tuberculosis*, vero cells and herpes simplex virus which all extracts showed insignificant activities [92]
28	*Drynaria sparsisora* (Desv.) T. Moore	Indonesia, Philippines, Thailand	LF, RZ	External, decoction	Rhizome: headache, fever, diarrhea, gonorrhea, swollen limbs, fever. Leaves: anti-vomiting, snake bite, eye infection [21,71,93]	
29	*Drynaria roosii* Nakaike	China	WP	Decoction	Deficient kidney, invigorate blood, heal wound, stop bleeding [21]	Compound **230** was isolated and the biotesting showed the highest stimulation toward UMR 106 cells (osteoblast) by 42.6% at a concentration of 1 µM [94]
30	*Drynaria propinqua* (Wall. ex Mett.) Bedd	Bhutan, India and Nepal	ST	Pills	Antidote and detoxifier especially when suffering from meat poisoning and other human-made poisons (*sbyar-dug*) [95]	
31	*Drynaria quercifolia* (L.) J.Sm.	Malaysia, Philippines, Indonesia, India	LF, RZ	Decoction, poultice	Swelling, fever (poultice leaves), haemoptysis, typhoid fever, ulcers, dyspepsia, artharlgia, diarrhea (decocted rhizome), inflammation, anthelmitic, cough, fever, phthisis, poultice of rhizome mixed with *Lannea coromandelica* (Houtt.) Merr.) to treat headache, hepatoprotective agent [21,22,96]	Compound **200** from the ethyl acetate fraction to be responsible for good antimicrobial activity [97]
32	*Lepisorus contortus* (Christ) Ching	Bhutan, India, China	LF	Powder	Heals bone fracture, burns, wounds and kidney disorders [98]	
33	*Loxogramme involuta* (D. Don) C. Presl	Indonesia	LF, WP	Smoked	Smoked with tobacco [18]	
34	*Loxogramme scolopendria* (Bory) Presley	Indonesia	LF	Smoked	Cigarette paper [99]	
35	*Microsorum fortunei* (T. Moore) Ching	Indonesia	WP	Decoction	Diuretic, promote blood circulation [49,51]	
36	*Microsorum punctatum* (L.) Copel.	India	LF	Juice	Diuretic, purgative, wounds [70]	
37	*Phlebodium aureum* (L.) J.Sm	Mexico	RZ	Decoction	Cough, fever, sudorific agents [57]	
38	*Phymatosorus scolopendria* (Burm. f.) Pic. Serm.	South-East Asia, Madagascar	RZ	Fragrance (external), poultice, decoction	Fragrance, gecko bites, accelerate childbirthRespiratory disorder [18,47]	Bronchodilator (**341**, in-vivo) [47]
39	*Platycerium coronarium* (Mull.) Desv.	Indonesia	LF	Poultice (salt added)	Thyroid edema, scabies [18,100]	
40	*Platycerium bifurcatum* (Cav.) C. Chr.	Indonesia	LF	Poultice (salt added)	Thyroid edema, scabies, fever, swelling [100,101]	
41	*Pleopeltis macrocarpa* (Bory ex Willd.) Kaulf.	South-Africa, Mexico, Guatemala	LF, RZ	Decoction	Sore throat, itches, cough, febrifuge [70,102]	
42	*Pyrrosia heterophylla* (L.) M.G. Price	India	WP	Poultice	Swelling, sprain, pain (cooling agent) [103]	
43	*Pyrrosia lanceolata* (L.) Farw.	Malaysia, South-Africa, Mexico	LF, WP	Juice, poultice, decoction	Dysentery, headache, colds, sore throats, itch guard [55,87]	
44	*Pyrrosia lingua* (Thunb.) Farw.	Japan, China, Indonesia, Pacific Islands	LF, WP	Decoction	Diuretic, anti-inflammation, analgesic, cough, stomachache, urinary disorder (diuretic agent) [87,104,105,106]	Antioxidant [107], inhibition effects on virus-induced CPE when SARS-CoV strain BJ001 [108]
45	*Pyrrosia longifolia* (Burm. f.) C.V. Morton	Indonesia, Pacific Islands	LF	Poultice (cold water)	Ease pains in labor [18,87]	
46	Pyrrosia petiolosa *(Christ) Ching*	China	WP	Decoction	Urinary tract infections, as diuretic [109]	
47	*Pyrrosia sheareri* (Baker) Ching	China	LF	Decoction	Bacillary dysentery, rheumatism [87,110]	Antioxidant [110]
	**Psilotaceae**					
48	*Psilotum nudum* (L.) P. Beauv.	India	LF, SP	Fresh, decoction	Diarrhea (infants), antibacterial, purgative [55]	
	**Pteridaceae**					
49	*Acrostichum aureum* L.	South-East Asia, Bangladesh, Fiji, China, Panama	LF, RZ	Eaten, decoction	Wounds, peptic ulcers and boils, worm infections, asthma, constipation, elephantiasis, febrifuge, chest pain, emollients [18,35]	Anti-implantation (EtOH extract, albino rats) [111], Anti-tumour (hella cells, MTT assay) [112], Antioxidant (DPPH), tyrosine inhibition (96-well microtitre), antibacterial activity [44,113], anti-cancer ((gastric: AGS; colon: HT-29 and breast: MDA-MB-435S) using the MTT assay) [114]
50	*Acrostichum speciosum* Willd.	South-East Asia			Thatch [18]	
51	*Taenitis blechnoides* (Willd.) Sw.	Malaysia	LF	Decoction	Postnatal protection [115]	
	***Selaginellaceae***					
52	*Selaginella tamariscina* (P.Beauv.) Spring	Nepal	WP, SP	Fresh (spore), decoction	Vermilion powder, prolapsed rectum, cough, bleeding piles, amenorrhea, antibacterial [57,116]	Anti-acne [117], thymus growth-stimulatory activity in adult mice (reversal of involution of thymus) and remarkable anti-lipid peroxidation activity [118]
	**Vittariaceae**					
53	*Vittaria elongata* Sw.	South-East Asia, Andaman	LF	Decoction	Rheumatism [57]	Cytotoxicity against two human cancer cell lines, lung carcinoma (NCI-H460) and central nervous system carcinoma (SF-268), antioxidant (DPPH) [119]
	**Non-Fern**					
	**Araceae**					
54	*Philodendron fragrantissimum* (Hook.) G.Don	Guyana, Suriname, Brazil	LF, RT	Decoction, external (leaves)	Inflammation, aphrodisiac, demulcent, diuretic [72]	
	**Aralliaceae**					
56	*Schefflera caudata* (Vidal) Merr. & Rolfe	Philippines	WP	Decoction	Tonic for women after birth [120]	
57	*Schefflera elliptica* (Blume) Harms.	South-East Asia, China, India	BK, LF, RT	Decoction, chewed, external	Bechic, vulnerary, toothache, aromatic bath, dropsy [120].	Antibacterial [121]
58	*Schefflera elliptifoliola* Merr.	Philippines	LF	Decoction	Tonic for woman after birth [120]	
59	*Schefflera oxyphylla* (Miq.) R.Vig.	Thailand, Malaysia, Indonesia	RT	Decoction	Sedative for frightened child, externally to treat fevers [120]	
60	*Schefflera simulans* Craib	Thailand, Malaysia	LF, RT	Decoction	Stomach problem, protective medicine after birth [120]	
	**Asclepiadaceae**					
61	*Asclopidae sp.*	Indonesia	LF, RT	Decoction	Promote blood circulation [71]	
62	*Dischidia acuminata* Costantin	Vietnam	WP	Decoction	Blenorrhoea, promote urination [19]	
63	*Dischidia bengalensis* Colebr.	Thailand	LT, RT	Latex (external), decoction (tonic)	Anthemintic (ringworm), tonic [122]	
64	*Dischidia imbricata* (Blume) Steud.	Indonesia	LF	Poultice	Gonorrhea, burns and wounds [25,123]	
65	*Dischidia major* (Vahl) Merr.	India, Thailand, Philippines, Malaysia, Brunei	LF, RT, WP	Decoction, chrused (external), chewed with areca catechu	Peptic ulcer, liver dysfunction (decocted leaves mixed with *Hoya kerii* Craib leaves and *Vanilla aphylla* Blume stem), fever (root), goiter (crushed leaves mixed with salt), cough (root mixed betel quid), wound and injuries, stomache [19,124,125]	
66	*Dischidia nummularia* R.Br.	Thailand, Indonesia	LF, LT, WP	Decoction, latex (external)	Wound, gonorrhea, sprue in children, cirrhosis [126]	
67	*Dischidia platyphylla* Schltr	Philippines	LF	Decoction	Putrefaction [19]	
68	*Dischidia purpurea* Merr.	Philippines	LF	Crushed leaves mixed with coconut oil applied as external poultice	Eczema, herpes [19,127]	
69	*Toxocarpus sp.*	Indonesia	LF	Decoction	Headache, fever, nervous system problem [71]	
	**Balsaminaceae**					
70	*Impatiens niamniamensis* Gilg (semi epiphytic)	Congo	LF	Poultice	Wounds, sores, pain [128]	Anti-hyperglicemic (Rat) [129]
71	**Convolvulaceace *(parasite)***					
72	*Cassytha filiformis L*	India, Taiwan, China, Vietnam, Malaysia, Philippines, Indonesia, Fiji, Africa, Central America.	WP, NT	Decoction	Cough, dysentery, diarrhea, intestinal problems, headache, malaria fever, nephritis, edema, hepatitis, sinusitis, gonorrhea, syphilis, skin ulcer, eczema, prevent haemoptysis. Parasite skin and scalp. Induce lactation (after still birth), promote hair growth, diuretic, vermifuge, laxative agent, saliva blood removal (childbirth) [19,130,131,132]	An *α*1-adrenoceptor antagonist (Rat thoracic aorta) [133], antiplatelet and vasorelaxing actions (Rabit platelet, aortic contraction) [134], anti-trypanosomal, citotoxicity [135], antioxidant [136]
73	*Cuscuta australis* R.Br.	Indonesia, Vietnam, China	WP, SD	Decoction, poultice	Whole plant: emollient, sedative, sudorific and tonic agents, urinary complaint. The seeds: sedative agent, diabetes, cornea opacity, acne, dandruff [137].	Cytotoxicity, antioxidant activity, and inhibitory effects on tyrosinase activity and melanin biosynthesis were estd. by using melanoma Clone M-3 [138]
74	*Cuscuta reflexa* Roxb.	India	WP	Decoction, poultice	Mixed with the twigs of *Vitex negundo* L. applied as fomentation on the abdomen of kwarsiokor children, fever, itchy [139,140]	Anti-viral [141,142], anti-HIV [143], analgesic, relaxant (ether extract) [144], antisteroidogenic activity (MeOH extract) [141], antibacterial activity [145], hair growth activity in androgen-induced alopecia [146], anti-inflammatory (murine macrophage cell line RAW264.7), anti-cancer (Hep3B cells by MTT assay) [147], antioxidant (etOAc extract, DPPH), anti-obesity (EtOAc extract) [148]
	**Clusiaceae**					
75	*Clusia grandiflora* Splitg. (hemi epiphyte)	Guyana, Suriname	RT	Decoction	Aphrodisiac [72]	Antibacterial [149]
76	*Clusia fockeana* Miq. (hemi epiphyte)	Guyana, Suriname	ST(Exudate)	Poultice	Snake bites, ulcers [72]	
	**Gesneriaceae**					
77	*Columnea nicaraguensis* Oerst.	Panama	ST, LF, WP	Decoction, maceration	Fever [150]	
78	*Columnea sanguinolenta* (Klotzsch ex Oerst.) Hanst.	Panama	ST, LF	Decoction	Dysmenorrhea [150]	
79	*Columnea tulae* Urb. var. *tomentulosa* (C.V. Morton) B.D. Morley	Panama	ST	Decoction	Fever [150]	
80	*Drymonia serrulata* (Jacq.) Mart.	Amazon	na	Not mentioned	Eczema [151]	Analgesic, anti-inflammatory [152]
81	*Drymonia coriacea* (Oerst. ex Hanst.) Wiehler	Amazon	na	Not mentioned	Toothache [151]	
	**Loganiaceae**					
82	*Fagraea auriculata* Jack. (semi epiphyte)	Indonesia	ST		Stem for stick [25]	Anti-inflammatory [153]
	**Loranthaceae *(parasite)***					
83	*Amyema bifurcata* (Benth.) Tiegh.	Australia	ST, LF	Decoction	Colds, fever, sores [154]	
84	*Amyema quandang* (Lindl.) Tiegh.	Australia	LF	Decoction	Fever [155]	
85	*Amyema maidenii* (Blakely) Barlow	Australia	FT	Decoction	Inflammation in the genital regions [156]	
86	*Dendrophthoe falcata* (L.f.) Ettingsh	India	WP	Decoction	Pulmonary tuberculosis, asthma, menstrual disorders, swellings, wounds, ulcers, strangury, renal and vesical calculi, aphrodisiac, astringent, narcotic, diuretic [157].	Wound healing activity was studied, antimicrobial activity and antioxidant activity [158]
87	*Dendrophthoe frutescens* L.	Indonesia	LF, WP	Drink (decoction)	Anti-inflammation, antibacterial [51]	
88	*Dendrophthoe incarnata* (Jack) Miq.	Malaysia	LF	Poultice	Mixed with *Curcuma longa* L and rice to make poultice to treat ringworm [159]	
89	*Dendrophthoe pentandra* (L.) Miq.	Indonesia, Malaysia, Thailand, Vietnam	LF, WP	Poultice, decoction	Sores, ulcers, other skins infections, protective medicine after childbirth, cough, hypertension, cancer, diabetes, tonsil problem [18,25,159,160]	Antioxidant (MeOH extract, DPPH), Tyrosinase activity [160]
90	*Taxillus umbellifer* (Schult. f.) Danser	Indonesia, Malaysia, Vietnam	RT, LF	Decoction drink, poultice	Fever, headache, wounds [159]	
91	*Erianthemum dregei* (Eckl. & Zeyh.) Tiegh.	Southern & Eastern Africa	BK	Mixed with milk	Powdered mixed with milk to treat stomach problems in children [161]	
92	*Loranthus globosus* Roxb	Malaysia, Indo-China	LF, ST, FT	Poultice (leaves), juice	Headache, expel afterbirth, cough [162]	Antimicrobial, cytotoxicity (brine shrimp) [163], toxicity (Evan’s rat) [164]
93	*Loranthus* spec div.	Indonesia	WP	Poultice, decoction	Ariola, varicella, diarrhea, ankylostomiasis, morbilli (gabag), cancer [25]	
94	*Macrosolen robinsonii* (Gamble) Danser	Vietnam	LF	Decoction	Enlarged abdomen (diuretic tea) [165]	
95	*Macrosolen cochinchinensis* (Lour.) Tiegh.	Malaysia, Indo-China	ST, LF	Decoction, juice, poultice	Expel after birth, headache, cough [165]	
96	*Scurrula atropurpurea* (Blume) Danser	Indonesia, Philippines	LF, ST, WP	Decoction	Mouthwash (gargled), cancer (breast, throat cancer), cowpox, chickenpox, diarrhea, hookworm, measles, hepatitis, and cancer [166,167,168]	Cancer cell invasion inhibitory effects [169,170]
97	*Scurrula ferruginea* (Jack) Danser	Malaysia	LF, WP	Decoction, poultice	Decocted whole plant (mixed with *Millettia sericea* (Vent.) Wight & Arnott) is used as bathing to relieve malaria, decocted leaves as protective medicine after childbirth, pounded leaves to treat wounds, snake bites [166]	Antiviral (HSV-1 and poliovirus) and cytotoxic activities on murine and human cancer lines (3LL, L1210, K562, U251, DU145, MCF-7) [171]
98	*Scurrula parasitica* L.	China, Vietnam	WP	Decoction	Swelling, back pains, numbness, soreness of limbs, hypertension, galactagogue, quieting uterus (no contraction), reducing lumbago, bone strengthening. [166]	Anti-cancer (flavonoids extract, Leukimia cell line HL-60) [172], NF-κB inhibition [173], recovery of cisplatin-induced nephrotoxicity [174], Antioxidant (extracts, DPPH) [175] anti-cancer (Polysacharide fraction, S180, K562 and HL-60 cell lines, MTT assay) [176], anti-obesity activity using porcine pancreatic lipase assay (EtOH extract, PPL; triacylglycerol lipase, EC 3.1.1.3)[177], neuroprotective activity (**168**, H_2_O_2_-induced oxidative damage in NG108-15 cells)[178], antibacterial (EtOH extract, MRSA) [179]
99	*Viscum aethiopicum* [sic]	Southern & Eastern Africa	LF	Decoction (tea)	Diarrhea [161]	
100	*Viscum capense* L.f.	Southern & Eastern Africa	ST, FT	Decoction, external	Wart, asthma, irregular menstruation, hemorrhage [161]	Antimicrobial activity (stems extract), Anticonvulsant activity (MeOH extract, albino mice) [180]
101	*Viscum pauciflorum* L.f.	Southern & Eastern Africa	WP	Decoction	Astringent [161]	
102	*Viscum rotundifolium* L.f.	Southern & Eastern Africa	WP	External	Wart [161]	Immunoassay (stem, aqueous extracts, T cell activity in ruminants) [181]
	**Melastomataceae**					
103	*Medinilla radicans* Blume		LF, RT	Leaves eaten to treat dysentery, adventitious roots applied as poultice to wound, young leaves to skin disorders	Dysentery, wound and skin disorders [123]	
104	*Pachycentria constricta* (Bl) Blume	Indonesia	TB	Tubers are boiled and eaten	Hemorrhoids [18,71]	
	**Moraceae**					
105	*Ficus annulata* Blume	Indonesia	LF, RT	Leaves decoction to treat fever, the root to treat Hansen diseases	Fever and Hansen diseases [168]	
106	*Ficus deltoidea* Jack	Indonesia, Malaysia, Thailand	LF, RT, FT	Drink (decoction), oitment	Leucorrhea, headache, fever, diabetes, high blood pressure, skin infection, aphrodisiac agent, ornament [71,182,183,184]	Toxicity (aqueous extract, rats) [185], anti-nociceptive [186], antioxidant (leaves aqueous extracs, redn. power of iron (III), superoxide anion (O2-) scavenging, xanthine oxidase (XOD), nitric oxide (NO·) and lipid peroxidn) [187], anti-melanogenic effect (extract, B16F1 melanoma cells, MTT assay) [188], anti-cancer [189], hypoglycemic activity (extract, rodents) [45,188] antimicrobial activity (extract) [190], Anti-inflammatory [191]
107	*Ficus lacor* Buch.-Ham.	India	BK, LT, BD, SD	Decoction, poultice	Decocted stem bark to treat gastric and ulcer, latex to treat boils (external), typhoid and fever (internal), decocted bud to treat ulcer, leucorrhoea, Seed as tonic for stomach disorder [157,192,193,194]	The medicated liquor has effects of relaxing muscles and tendons, activating collateral flow, promoting blood circulation, dispelling blood stasis, expelling wind, removing dampness, and relieving pain [195]
108	*Ficus natalensis* Hochst. (semi epiphytic, secondary terrestrial)	Uganda, Tanzania, Senegal, West Africa, South Africa,	LF, LT, RT, BK	Decoction, poultice	Root was used to treat lumbago, headache, arthritis, cataract and cough, Leaves were used to treat snakes bite, malaria, dysentery, ulcers, wounds and used as septic ears [196]	Antibacterial, antimalarial, and/or antileishmania activities were obsd. in some crude extracts., and five of these exts. showed a significant cytotoxicity against human tumor cells [41]
109	*Ficus parietalis* Blume	Vietnam, Thailand, Malaysia, Indonesia	RT	Decoction	Stomach-ache [184]	
110	*Ficus pumila* L.	Vietnam	FT, LF, LT	Drink (decoction)	Diarrahea, hemaroid, rheumatic, anemia, haematura, dysentery, dropsy, galactoge, tonic for impotence, lumbago, anthelmintic agent, externally used to treat carbuncles [184]	Against T-cell leukemia [197], antimicrobial [198]
111	*Poikilospermum suaveolens* (Blume) Merr.	Indonesia, Thailand	BK	Decoction	Water from the stem for drink, aide the secretion of waste products from the vagina, pain, numbness, stomach ulcer [25,199,200]	Anti-viral (MeOH extract) [201]
	**Orchidaceae**					
112	*Acampe carinata* (Griff.) Panigrahi	Himalaya, Nepal	WP	Decoction	Rheumatism, sciatica, neuralgia, beneficial in secondary syphilis and uterine diseases [202]	
113	*Acriopsis liliifolia* (J.Koenig) Seidenf.	Malaysia	LF, RT	Decoction of the roots and leaves	Fever [203]	
114	*Anoectochilus formosanus* Hayata	Taiwan	WP	Decoction	Fever, anti-inflammatory agent, diabetes, liver disorder, chest and abdominal pain [204]	Anti-inflammatory (water extract, rat paw), hepatoprotective (water extract, rat, SGOT-OPT) [205], anti-hyperliposis (**414**, rat induced) [206], ameliorative effect (water extract, ovariectomised rat) [207], antioxidant (water extract, DPPH) [208], anti-hyperglycemic (water extract, diabetic rats induced by streptozotocin) [209], anti-cancer (extracts, breast cancer MCF-7 cell) [210], liver regeneration (extract, rat) [211,212], Hepatoprotective (**414**, CCl_4_ induced rat) anti-inflammatory (**414**, lps stimulate mice) [213,214], anti-cancer (polysaccharide water extract, protate cancer cell lin PC3) [215]
115	*Anoectochilus roxburghii* (Wall.) Lindl.	Taiwan, China, Japan	WP	Decoction	Fever, snake bite, lung and liver diseases, hypertension, child malnutrition [216]	Hypoglycemic effect (**414**, streptozotocin (STZ) diabetic rats) [217], hypoglycemic and antioxidant effects (water extract, alloxan-induced diabetic mice, DPPH) [218]
116	*Ansellia africana* Lindl.	Southern & Eastern Africa	PD, ST, ST, RT	Decoction	Pedi is used to treat cough, the stem is used as aphrodisiac, used as emetic agent [161]	
117	*Bulbophyllum kwangtungense* Schltr.	China, Japan	TB	Tonic	To treat pulmonary tuberculosis, promote body liquid production, reduce fever, hemostatic agent [219]	Anti-tumor activities (**456**, **457**, **458**, against HeLa and K562 human tumor cell line) [220]
118	*Bulbophyllum odoratissimum* (Sm.) Lindl. ex Wall.	China, Burma, Vietnam, Thailand, Laos, Nepal, Bhutan, India	WP	Decoction	To treat pulmonary tuberculosis, chronic inflammation and fracture [221]	Anti-tumor (bibenzyl, inhibiting NO microphage) [221,222], anti-cancer (**225**, **470**, **471**, **475**, **476**, **478**, **479**, **482**, **484**, human leukaemia cell lines K562 and HL-60, human lung adenocarcinoma A549, human hepatoma BEL-7402 and human stomach cancer SGC-790) [223], anti-cancer (human leukemia cell lines K562 and HL-60, human lung adenocarcinoma A549, human hepatoma BEL-7402 and human stomach cancer cell lines SGC-7901) Anti-cancer (**473** and **474**, human leukemia cell lines K562 and HL-60, human lung adenocarcinoma A549, human hepatoma BEL-7402 and human stomach cancer SGC-7901) [224]
119	*Bulbophyllum vaginatum* (Lindl.) Rchb.f.	Malaysia	WP	Juice	Juice of the plant is instilled in the ear to cure earache [130]	
120	*Catasetum barbatum* (Lindl.) Lindl.	Japan, Guiana, Paraguayan	WP	Decoction	Febrifuge, anti-inflammatory [46]	Anti-inflammatory (**505**, rat) [225]
121	*Coelogyne sp*	Indonesia	RT	Decoction	Headache, fever [71]	
122	*Cymbidium aloifolium* (L.) Sw.	Thailand, Vietnam	LF	Decoction (internal), juice from heated or crushed leaves.	Otitis media, colds, irregular periods, arthritis, sores, burns, tonic [226]	Antinociceptive, anti-inflammatory (EtOH extract, mice) [227]
123	*Cymbidium canaliculatum* R.Br	Australia	PdB	Chewed, poultice	Dysentery, boils, sores, wounds, itschy skin, fractured arms over the break [154,228]	
124	*Cymbidium ensifolium* (L.) Sw	Taiwan, Vietnam	LF, RT, FL, WP, RT	Decoction	Diuretic agent (leaves), pectoral agent (root), eye problem (flower), cough, lung, gastrointestinal problems and sedative [226]	
125	*Cymbidium goeringii* (Rchb.f.) Rchb.f.	Japan, China, Korea, Thailand, Vietnam, India	WP	Decoction	Hypertension, diuretic agent [229]	Anti-inflammatory (**478**, RAW 264.7 cells) [230], anti-hypertensive (**515**, rat), diuretic activity (**515**, rats) [229]
126	*Cymbidium madidum* Lindl.	Australia	PdB	Chewed	Dysentery [154]	
127	*Dendrobium affine* (Decne.) Steud.	Australia	PdB	Poultice, external	Chrushed pseudobulbs (sticky) is applied to itchy skins, boils, infected skin lesion, minor burns [154]	
128	*Dendrobium aloifolium* (Blume) Rchb.f.	South East Asia	LF	Poultice	Headache [18]	
129	*Dendrobium amoenum* Wall. ex Lindl.	China	LF	Dried and ground	Skin diseases [231]	Antioxidant (**519**, NBT), antibacterial (**519**, diffusion) [231]
130	*Dendrobium chryseum Rolfe*	Australia	LF	Decoction	Diabetes [232]	Antioxidant (**526**, **530**, **532**, DPPH) [233]
131	*Dendrobium candidum* Wall. ex Lindl.	China	LF	Decoction	Diabetes [234]	Inhibitory effect of atropine on salivary secretion (extracts, rabbit) [235], anti-hyperglicemic (extract, streptozotocin-induced diabetic (STZ-DM) rats) [234], antioxidant (polysaccharide, 10-phenanthroline-Fe^2+^-H_2_O_2_ systems and ammonium peroxydisulfate/*N*,*N*,*N*’,*N*’-tetra-methylethanediamine systems) [236] antioxidant (**555**, **556**, DPPH) [237], antioxidant (**558**, **559**, **560**, DPPH) [238], anti-tumor (soluble polysacharride, human neuroblastoma (SH2SY5Y) induced by SPD was observed and analyzed by Hoechst stain method) [239]
132	*Dendrobium canaliculatum* var. foelschei (F.Muell.) Rupp & T.E.Hunt	Australia	PdB	Poultice, external	Chrushed pseudobulbs (sticky) is applied to infected skin and cuts [154]	
133	*Dendrobium crumenatum* Sw.	Malaysia, Indonesia	LF, PdTB	Leaves pounded, bulbs heated to produce juice and applied as external uses	Acne (leaves), infected ears (pseudo-tubers) [240,241]	Antimicrobial [242]
134	*Dendrobium chrysanthum* Wall. ex Lindl.	China	LF	Dried and ground	Skin diseases, immune regulator, anti-pyretic, improve eyesight [243,244]	Anti-inflammation (**590**, macrophages were harvested from 2-month-old male C57BL/6J mice) [244]
135	*Dendrobium densiflorum* Lindl.	China	LF	Tonic	Promote body fluid production [245]	
136	*Dendrobium faciferum* J.J.Sm	Indonesia	ST	Dried	For twist work (craft) [246]	
137	*Dendrobium fimbriatum* Hook.	Japan, China	LF	Decoction, paste	Promote body fluid production, set fractured bone (paste) [247]	Antioxidant (water-soluble crude polysaccharide (DFHP), DPPH) [248]
138	*Dendrobium loddigesii* Rolfe	China	LF	Decoction	Promote body fluid production, reduce fever, nourish the stomach., anti-cancer agent [249]	Inhibitors of Na^+^, K^+^-ATPase of rat kidney (**607**, **608**) [250], antiplatelet aggregation activity (**479**, **523**, **606**, rabit platelet) [251], antioxidant (DPPH), anti NO production (activated macrophages-like cell line, RAW264.7) [252]
139	*Dendrobium moniliforme* (L.) Sw.	China, Taiwan	ST	Decocted dried stem	Anti-pyretic, analgesic, aphrodisiac, stomachic, tonic agents [253]	Anti-inflammatory (**552**, RAW 264.7 cells) [254], hypoglicemic (polisaccharide, mice) [255], antioxidant (polisacharide) [256]
140	*Dendrobium moschatum* (Buch.-Ham) S.w	Nepal	LF	Juice	Cure earache [257]	
141	*Dendrobium nobile* Lindl.	China, Indonesia	WP	Tonic	Fever, reduce mouth dryness, aphrodisiac, promote body fluid production, nourish stomach, anorexia, lumbago, impotence [240,258,259,260,261]	Immunomodulatory activity (**656**, **660**, **661**, **662**, **663**, lymphocyte proliferation test MTT test) [262,263], antioxidant (**478**, **523**, **524**, **528**, **584**, **641**, **672**, **673**, **674**, DPPH) anti-NO (**478**, **523**, **524**, **528**, **584**, **641**, **672**, **673**, **674**, murine macrophage-like cell line RAW 264.7) [264], antioxidant (water-soluble polysaccharide (DNP), DPPH) [265], antimicrobial (Extracts), antitumour (extracts, Dalton’s lymphoma ascites (DLA) cells w), induction of in vitro lipid peroxidation (extracts, TBARS) [266], NO inhibition (**475**, **523**, **542**, **632**, **633**, **634**, **665–671**, murine macrophage RAW 264.7 cells) [267], anti-tumor (polisachacaride extracts, sarcoma 180 in vivo and HL-60)[268]
142	*Dendrobium pachyphyllum* (Kuntze) Bakh.f.	Indonesia	WP	Decoction	Hydropsy [246]	
143	*Dendrobium purpureum* Roxb.	Indonesia, Malaysia	LF	Crushed and heated to make poultice	Nail fungal infection [240]	
144	*Dendrobium salaccense* (Blume) Lindl.	Indonesia	LF	Fragrance	Fragrance [246]	
145	*Dendrobium teretifolium* R.Br.	South-Pacific Island	LF	Decoction	Severe headache, other pains [269,270]	
146	*Dendrobium catenatum* Lindl.	China	LF	Decoction	Anxiety and panic [271]	
147	*Dendrobium utile* J.J.Sm.	Indonesia	ST	Dried	Twist work [246]	
148	*Dichaea muricata* (Sw.) Lindl.	Central, South American	LF	Decoction (wash)	Eye infection [260]	
149	*Eulophia speciosa* (R.Br.) Bolus	Indonesia	RT	Decoction	Analgesic [246]	
150	*Epidendrum strobiliferum* Rchb.f.	China, Korea	ST	Infusion, decoction	Analgesic [272]	Analgesic (**676**, **677** exhibited notable analgesic action at 3 mg/kg, causing 86 and 83% inhibition of abdominal constriction, respectively [272], antinociceptive effect (MeOH extract, methanolic ext. (ME) [273]
151	*Epidendrum rigidum* Jacq.	Mexico, North Sudamerica, Antilles	ST	Infusion, decoction	Replenish body fluid [274]	Phytotoxin (chloroform-methanol extract) [274]
152	*Mycaranthes pannea* (Lindl.) S.C.Chen & J.J.Wood	Vietnam, Malaysia	WP	External, medicinal bath	Medicinal bath to treat ague and malaria fever, fractures, bruises, skin complaints, dislocated joint to relieve severe pain, swelling, dislocation and fracture [123,275,276]	
153	*Eriopsis biloba* Lindl.	America	ST	Poultice	Sore gums and mouth membranes [260]	
154	*Grammatophyllum scriptum* (L.) Blume	Indonesia, Thailand	BL, SD, ST	Poultice	Pseudo bulb mixed with curcuma and salt applied to sores and abdomen to expel worms, to treat dropsy and aphthae, seeds mixed with food to treat dysentery, aphthae, crushed plant mixed with rice liquor to treat snake bite, scorpions’ and centipedes’ stings [246,277]	
155	*Jumellea fragrans* (Thouars) Schltr.	Madagascar	LF, ST	Decoction	Anti-spasmodic, anti-asthmatic agents, mixed leaves of *Ziziphus mauritana*, *Mussaenda arcuate* to treat eczema (deecotion), mixed with *Eugenia uniflora* to treat diarrhea [24]	
156	*Liparis condylobulbon* Rchb.f.	Indonesia	PdB, LF	Chewing, external	Intestinal complaints and constipation. (eastern Sulawesi, ambon), tormina, abscess [246,278]	
157	*Liparis nervosa* (Thunb.) Lindl.	China, Thailand, Malaysia	WP	Decoction, external	Stop internal/external bleeding, treat snake bites [278]	
158	*Neottia ovata* (L.) Bluff & Fingerh.	Spain	TB	Tincture	Stomach diseases [279]	Anti-viral (extract, SARS-CoV Frankfurt 1 strain [280]
159	*Masdevallia uniflora* Ruiz & Pav.	Mexico, south America	WP	Decoction	Facilitate urination (pregnant women), reduce bladder inflammation [260]	
160	*Camaridium densum* (Lindl.) M.A.Blanco	Mexico	WP	Decoction	Analgesic, relaxant agents [281]	Spasmolytic activity (**667**, **690**, **693**, **694**, **695**, Wistar rat) [37], antinociceptive activity (extract, mice) [281]
161	*Nidema boothii* (Lindl.) Schltr.	Malaysia	WP	Decoction	Relaxant agent [282]	Spasmolytic effects (**471**, **478**, **488**, **508**, **671**, **696**, **697**, **699**, **700**, **702**, guinea ileum pig model) [282]
162	*Oberonia lycopodioides* (J.Koenig) Ormerod	Malaysia	LF	Poultice	Boils [123,283]	
163	*Oberonia mucronata* (D.Don) Ormerod & Seidenf.	China, Vietnam	WP	Decoction	Rheumatism, promote blood circulation, inflammation of the bladder/ureter, bruises and fractures, detoxicant, diuretic agent [284]	
164	*Erycina pusilla* (L.) N.H.Williams & M.W.Chase	Mali	WP	Decoction	Lacerations [260]	
165	*Otochilus lancilabius* Seidenf.	Bhutan, Nepal, India, China (Tibet), Laos and Vietnam	WP	Pills	Antiemetic, febrifuge for stomach inflammation (*bad-tshad*), and allays hyperdipsia and dehydration [23]	
166	*Phragmipedium pearcei* (Rchb.f.) Rauh & Senghas	South America	WP	Decoction	Stomachache [260]	
167	*Pholidota articulata* Lindl.	Himalaya, Nepal	WP		Whole plant: bone fractures [202]	
168	*Pholidota chinensis* Lindl.	China, India	PdB	Tincture	Scrofula, toothache, stomachache, chronic bronchitis, duodenal ulcer [285]	Antioxidant (**475**, **539**, **667**, **670**, **671**, **711**, **712**, **717**, **722**, **723**, **726**, (DPPH), anti-inflammatory (**475**, **539**, **667**, **670**, **671**, **711**, **712**, **717**, **722**, **723**, **726**, inhibitory activity on NO production from activatedmacrophage-like cell line, RAW 264.7)[286], antioxidant (**715**, **741**, **742**, **746**, **747**, **749**, **750**, DPPH), anti-inflammatory (as above, inhibitory activity on NO production from activated macrophages-like cell line, RAW 264.7) [285]
169	*Renanthera moluccana* Blume	Indonesia	WP	Ornament	Ornament [246]	
170	*Rhynchostylis retusa* (L.) Blume	Himalaya, Nepal, India	LF		Rheumatic, hepaoprotective agent [96,202]	
171	*Scaphyglottis livida* (Lindl.) Schltr.	Mexico	WP	Decoction	Analgesic, anti-inflammatory agents [281,287]	Spasmolytic (**471**, **475**, **714**, **754**, **755**, rat ileum rings) [288], antinociceptive (extracts, male mice ICR) [281], acute toxicity (extract, male mice ICR) [287]
172	*Vanda tessellata* (Roxb.) Hook. ex G.Don	India, Sri Lanka, Burma	LF, RT, FL	Leaves pounded to make juice, paste, extract (alcoholic) of the root and flower	Fever (as paste), otitis (dropped juice), the root to treat bronchitis, rheumatic, dyspepsia, sciatica, inflammation, otitis, nervous problem, fever and as aphrodisiac, laxative, tonic (for liver) agent [140,289,290,291]	Cholinergic activity (glycoside fraction), anti-arthritic (extract, albino rat) [292], anti-inflammatory (extract), antidiabetic (extract, rat) [291,293]
173	*Papilionanthe teres* (Roxb.) Schltr.	Indonesia	WP	Ornament	Ornamental [294]	Anti-aging (**758**, **759**, HaCaT cytochrome C oxidase) [295]
174	*Vanilla griffithii* Rchb.f.	Indonesia	WP	Eaten	Edible [294]	
175	*Vanilla planifolia* Jacks. ex Andrews	Indonesia, Mexico	FT, STh	Decoction	Fever, rheumatism, hysteria, increase energy and muscular system [25,259,294]	Antimicrobial activity (extract) [296]
	**Piperaceae**					
176	*Peperomia galioides* Kunth	Peru	WP	Poultice (external), drink (internal)	Chrused plant is used to treat wounds, cuts, plant juice is used to treat gastric ulcers [297]	Antibacterial (oil) [298,299]
177	*Piper retrofractum* Vahl	Indonesia	FT, RT	Drink (decoction)	Anticonvulsion, antivomiting, diarrhea, dysentery, constipation, headache [300]	Anti-convulsan (**776**, mice) [301], cytotoxicity (extract, **779**) [302], anti-platelet aggregation (extract) [303], anti-vector (extract, mosquito larvae) [304,305], antioxidant (**228**, **283**, **334**, **574**, **771**, **772**, **782**, **783**, DPPH) [306], antileishmanial activity (extracts, leishmania donovani) [307], anti-obesity (**776**, **777**, C57BL/6J mice) [308]
	**Rubiaceae**					
178	*Hydnophytum formicarum* Jack	Indonesia, Philippines, Thailand	TB	Poultice, decoction, powder	Poultice to treat swelling, headache, decoction to treat liver, intestinal complaints, powder as anthelmintic, heart tonic, antidiabetic agent and to treat skin, bone, knee, ankle, lung diseases [278]	Anti-tumor (extracts, against human tumor cell lines, HeLa and A549) [309], xanthine oxidase inhibitory (MeOH extract, assayed spectrophotometrically under aerobic conditions [310], antimicrobial, cytotoxicity (**226**, **786**, **787**, against HuCCA-1 and KB cell lines) [311], trigger cytochrome C release in treated MCF-7 cell (**786**, ELISA) [312], anti-cancer (**786**, the human breast carcinoma cell line MCF-7) [313]
179	*Myrmecodia tuberosa* Jack	Indonesia	PT	Drink (decocted)	Swelling, headache [18,71,314]	Immunomodulatory effect (EtOH fractions) [315]
180	*Myrmecodia pendens* Merr. & L.M.Perry	Papua	PT	Decoction	Rheumatism, headache, renal problems, tumor [316]	
	**Sterculiaceae**					
181	*Scaphium macropodum* (Miq.) Beumée ex K.Heyne (hemi-epiphyte)	Indonesia	RT	Drink (decoction)	Nervous system problem [71]	
	**Verbenaceae**					
182	*Premna parasitica* Blume	Indonesia	LF	Drink (decoction)	Fever [25]	
	**Viscaceae**					
183	*Viscum articulatum* Burm.f.	Cambodia, India, Taiwan, China	WP	Poultice, decoction	Decoction to treat bronchitis, skin tumour, neuralgia, arthritis and as tonic, sedative, febrifuge, crushed plant to treat cut [317]	Toxicity (extract, mice) [318], anti-tumor (**820**, MTT assay) [319], anti-inflammatory (1234718, superoxide inhibition) [320], cytotoxicity and anti-HIV-1 activity (shown by isolated compounds including **801**, **804**, **803**, **813**, **814**, **815**, **824**, **828**); MDAMB-435 and Hela cells, HIV-1ШB-infected C8166 cells) [321], anti-nephrotoxic (**127**, gentamicin-induced renal damage in Wistar rats) [322], antioxidant, anti-inflammatory (**810**, **811**, **812**, **822**, **825**, **829**, **830**, **831**, **832**, **833**, **834**, DPPH, NO production and cell viability assay. The murine macrophage cell line RAW264.7) [323], diuretic activity (MeOH extract, male rats) [324], antiepileptic activity (MeOH exctract, rat) [325], anti-hypertension (glucocorticoid-induced hypertension, *Nω*-nitro-l-arginine methyl in rats) [326,327], antioxidant (polisacharide fraction, DPPH) [328]
184	*Viscum ovalifolium* DC.	Cambodia, Malaysia	LF, WP	Poultice, external	Leaves (poultice) to treat neuralgia, as herbal bath to treat fever in children, ash mixed with sulphur, coconut oil to treat pustular itches [329]	
	**Zingiberaceae**					
185	*Hedychium ongi cornotum* Griff.	Indonesia	RZ, RT	Drink (decoction)	Rhizome is used to treat syphilis; root is used to treat worm [25]	

**Note:** na: not mentioned; ST: stem, PT: pith; TB: tuber; SP: spore; BK: bark; LT: latex; NT: nutmeg; SD: seed; FT: fruit; BD: buds; PD: pedi; PdB: pseudobulbs; FL: flower; PdTB: pseudotuber; BL: bulbs: STh: sheath; WP: whole; LF: leaf; RT: root; RZ: rhizome.

**Table 2:** Phyctochemical constituents of epiphytic medicinal plants.

No	Epiphyte Species	Constituents
	**Fern species**	
	**Adiantaceae**	
1	*Adiantum caudatum* L., Mant	16-hentriacontanone **1**, 19*α*-hydroxyferna-7,9(11)-diene **2**, 29-norhopan-22-ol **3**, 3*α*-hydroxy-4*α*-methoxyfilicane **4**, 8*α*-hydroxyfernan-25,7*β*-olide **5**, adiantone **6**, filic-3-ene **7**, hentriacontane **8**, isoadiantone **9**, quercetin-3-*O*-glucoside **10**, *β*-sitosterol **11**, *β*-sitosterol **11**, *β*-sitosterol glucoside **12** [330,331,332]
	**Aspleanceae**	
2	*Asplenium nidus* L.	(-)-epiafzelechin 3-*O*-*β*-d-allopyranoside **13**, homoserine **14** [333]
	**Blechnaceae**	
3	*Stenochlaena palustris* (Burm. F.) Bedd.	1-*O*-*β*-D-glucopyranosyl-(2S*,3R*,4*E*,8*Z*)-2-*N*-[(2*R*)-hydroxytetracosanoyl]octadecasphinga 4,8-dienine **15**, 3-formylindole **16**, 3-oxo-4,5-dihydro-*α*-ionyl-*β*-d-lucopyranoside **17**, kaempferol 3-*O*-*β*-d-glucopyranoside **18**, kaempferol 3-*O*-(3′,6′-di-*O*-*E*-*p*-coumaroyl)-*β*-d-glucopyranoside **19**, kaempferol 3-*O*-(3′-*O*-*E*-*p*-coumaroyl)-(6′-*O*-*E*-feruloyl)-*β*-d-glucopyranoside **20**, kaempferol 3-*O*-(3′-*O*-*E*-*p*-coumaroyl)-*β*-d-glucopyranoside **21**, kaempferol 3-*O*-(6′-*O*-*E*-*p*-coumaroyl)-*β*-d-glucopyranoside **22**, lutein **23**, stenopaluside **24**, stenopalustrosides A–E **25–29**, *β*-sitosterol-3-*O*-*β*-d-glucopyranoside **30** [334,335]
	**Davalliaceae**	
4	*Araiostegia divaricata* (Blume) M. Kato	(-)-epicatechin 3-*O*-*β*-d-(2”-*O*-vanillyl)allopyranoside **31**, (-)-epicatechin 3-*O*-*β*-*D*-(2′-trans-cinnamoyl)allopyranoside **32**, (-)-epicatechin 3-*O*-*β*-D-(3”-O-vanillvl)allopyranoside **33**, (-)-epicatechin 3-*O*-*β*-d-(3′-*trans*-cinnamoyl)allopyranoside **34**, (-)-epicatechin 3-*O*-*β*-d-allopyranoside **35**, (-)-epicatechin 3-*O*-*β*-d-allopyranoside **35**, (+)-catechin 3-*O*-*β*-allopyranoside **36**, 24-norferna-4 (23) **37**, 4*β*-carboxymethyl-(-)-epicatechin **38**, 4*β*-carboxymethyl-(-)-epicatechin methyl ester **39**, 4*β*-carboxymethyl-(-)-epicatechin potasium **40**, 9(11)-diene **41**, cyanin **42**, davallic acid **43**, epiafzelechin-(4*β*→8)-epicatechin 3-*O*-*β*-d-allopyranoside **44**, epicatechin-(4*β*→6)-epicatechin-(4*β*→8)-epicatechin-(4*β*→6)-epicatechin-D-glucooctono-*δ*-lactone enediol **45**, epicatechin-(4*β*→8)-4*β*-carboxymethylpicatechin **46**, hop-21-ene **47**, monardein **48**, pelargonin **49**, procyanidin B-2 3”-O-*β*-d-allopyranoside **50**, sodium salts **51** [59,60,336,337,338,339,340]
5	*Davallia solida* (G. Forst.) Sw.	18-diene **52**, 18-diene **52**, 19*α*-hydroxyfernenes **53**, 19*α*-hydroxyfilic-3-ene **54**, 2-C-*β*-d-glucopyranosyl-1,3,6,7-tetrahydroxyxanthone **55**, 2-C-*β*-d-xylopyranosyl-1,3,6,7-tetrahydroxyxanthone **56**, 2-C-*β*-d-xylopyranosyl-1,3,6,7-tetrahydroxyxanthone **56**, 30-*O*-*p*-hydroxybenzoylmangiferin **57**, 3-*O*-*p*-hydroxybenzoylmangiferin **58**, 40-*O*-phydroxybenzoylmangiferin **59**, 4-*O*-*β*-d-glucopyranosyl-2,6,4′-trihydroxybenzophenone **60**, 4*β*-carboxymethyl-(-)-epicatechin **38**, 4*β*-carboxymethyl-(-)-epicatechin methyl ester **39**, 60-*O*-*p*-hydroxybenzoylmangiferin **61**, eriodictyol **62**, eriodictyol-8-*C*-*β*-d-glucopyranoside **63**, fena-9(11) **64**, fern-7-en-19*α*-ol **65**, fern-9(11)-en-19*α*-ol **66**, ferna-7 **67**, filic-3-en-19*α*-ol **68**, filica-3,18,20-triene **69**, filica-3,18-diene **70**, icariside E3 **71**, icariside E5 **72**, mangiferin **73** [66,68,338,341,342]
	**Lycopodiaceae**	
6	*Huperzia carinata* (Desv. ex Poir.) Trevis	carinatumins A, B, and C **74**, **75**, **76** [74]
7	*Huperzia phlegmaria* (L.) Rothm	14*β*,21*α*,29-trihydroxyserratan-3*β*-yl dihydrocaffeate (lycophlegmariol D) **77**, 21*α*,24-dihydroxyserrat-14-en-3*β*-yl 4-hydroxycinnamate (lycophlegmariol C) **78**, 21*β*,24,29-trihydroxyserrat-14-en-3*β*-yl dihydrocaffeate (lycophlegmariol B) **79**, 21*β*,29-dihydroxyserrat-14-en-3*α*-yl dihydrocaffeate (lycophlegmariol A) **80**, 21*β*-hydroxy-serrat-14-en-3*α*-ol **81**, 21*β*-hydroxy-serrat-14-en-3*α*-yl acetate **82**, 8,11,13-abietatriene-3*β*,12-dihydroxy-7-one (margocilin) **83**, 8-deoxy-13-dehydroserratinine **84**, 8-deoxyserratinidine **85**, acrifoline **86**, annotine **87**, annotinine **88**, dihydrolycopodine **89**, epidihydrofawcettidine **90**, fawcettidine **91**, huperzine A **92**, lycododine **93**, lycoflexine **94**, lycophlegmarin **95**, lycophlegmarin **95**, lycophlegmarine **96**, lycophlegmine **97**, lycopodine **98**, malycorin A **99**, malycorins B, C **100**, **101**, *N*,*N*′-dimethylphlegmarine **102**, phlegmanol A–E **103–107**, phlegmaric acid **108**, *α*-obscurine **109**, *β*-obscurine **110** [77,343,344,345,346,347,348]
8	*Huperzia megastachya* (Baker) Tardieu	21-*epi*-serratenediol **111**, 21-*epi*-serratenediol-3-acetate **112**, lycoclavanol **113**, megastachine **114**, phlegmanol-D **115**, serratenediol **116**, serratenediol-3-acetate **117**, serratenonediol diacetate **118**, tohogenol diacetate **119** [349,350]
9	*Nephrolepis biserrata* (Sw.) Schott	1*β*,11*α*-diacetoxy-11,12-epoxydrim-7-ene **120**, 1*β*,3*β*,11*α*-triacetoxy-11,12-epoxydrim-7-ene **121**, 1*β*,6*α*,11*α*-triacetoxy-11,12-epoxydrim-7-ene **122**, sequoyitol **123** [339,351]
	**Oleandraceae**	
10	*Nephrolepis cordifolia* (L.) C. Presl	fern-9(11)-ene **124**, hentriacontanoic acid **125**, myristic acid octadecylester **126**, oleanolic acid **127**, sequoyitol (patent) **123**, triacontanol **128**, *β*-sitosterol **11** [352,353]
	**Opioglossaceae**	
11	*Botrychum lanuginosum* Wall.ex Hook & Grev.	(6′-*O*-palmitoyl)-sitosterol-3-*O*-*β*-d-glucoside **129**, 1-*O*-*β*-D-glucopyranosyl-(2*S*,3*R*,4*E*,8*Z*)-2-[(2*R*-hydroxy hexadecanoyl) amino]-4,8-octadecadiene-1, 3-diol **130**, 30-nor-21*β*-hopan-22-one **131**, apigenin **132**, *β*-sitosterol **133**, daucosterol **134**, luteolin **135**, luteolin-7-*O*-glucoside **136**, thunberginol A **137** [354]
	**Polypodiaceae**	
12	*Drynaria roosii* Nakaike	kaempferol 3-*O*-*β*-d-glucopyranoside-7-*O*-*α*-l-arabinoside **138**, (2*R*)-naringin **139**, (2*S*)-narigenin-7-*O*-*β*-d-glucoside **140**, kaemperol 3-*O*-*α*-l-rhamnosyl-7-*O*-*β*-d-glucoside **141**, luteolin-7-*O*-*β*-d-neohesperidoside **142**, maltol glucoside **143**, (-)-epicatechin **144**, 12-*O*-caffeoyl-12-hydroxyldodecanoic acid **145**, xanthogalenol **146**, naringenin **147**, kushennol F **148**, sporaflavone G **149**, kuraninone **150**, leachianone A **151**, 8-phenylkaempferol **152**, kaempferol **153**, chiratone **154**, fern-9(11)-ene **155**, hop-22(29)-ene **156**, isoglaucanone **157**, dryocrassol **158**, dryocrassol acetate **159**, (+)-afzelechin-3-O-*β*-allopyranoside **160**, (+)-afzelechin-6-C-*β*-glucopyranoside **161**, 4*α*-carboxymethyl-(+)-catechin methyl ester **162**, (-)-epiafzelechin-(4β→8)-(-)-epiafzelechin-(4*β*→8)-4β-carboxymethyl-(-)-epiafzelechin methyl ester **163**, (-)-epiafzelechin-(4*β*→8)-4*β*-carboxymethyl-(-)-epicatechin methyl ester **164**, (-)-epiafzelechin-(4*β*→8)-4α-carboxymethy-(-)-epiafzelechin ethyl ester **165**, (-)-epiafzelechin-3-*O*-*β*-d-allopyranoside **166**, (-)-epicatechin-3*-O*-*β*-d-allopyranoside **167**, (+)-catechin **168**, 4*β*-carboxymethyl-(-)-epiafzelechin methyl ester **169**, 4*β*-carboxymethyl-(-)-epiafzelechin **170**, (-)-epiafzelechin-(4*β*→82→*O*→7)-epiafzelechin-(4*β*→8)-epiafzelechin **171**, (-)-epiafzelechin **172**, (-)-epiafzelechin-(4*β*→8)-4*β*-carboxymethyl-epiafzelechin methyl ester **173**, epicatechin-(4*β*→8)-epicatechin **174**, (+)-afzelechin **175**, (+)-epicatechin-3-*O*-*β*-d-allopyranoside **176**, (-)-epicatechin-8-*C*-*β*-d-gluclopyranoside **177**, (-)-epiafzelechin-5-*O*-*β*-d-allopyranoside **178**, drynachromoside A **179**, drynachromoside B **180**, fortunamide **181**, curcumine **182**, demethoxycurcumine **183**, bisdemethoxycurcumine **184**, bavachinine **185**, isobavachalcone **186**, (-)-epicatechin **144**, liquiritine **187**, bakuchiol **188**, protocatechuic acid **189**, (*R*)-5,7,3′,5′-tetrahydroxy-flavonone 7-*O*-neohesperidoside **190**, (2*S*)-5,7,3′,5′-tetrahydroxyflavonone 7-*O*-*β*-d-glucopyranoside **191**, 5,7,3′,5′-tetrahydroxflavanone **192**, 3′-lavandulyl-4-methoxy-2,2′,4′,6′-tetrahydroxyylcalcone **193**, 5,7-dihydroxychromone-7-*O*-*β*-d-glucopyranoside **194**, 5,7-dyhidroxychromone-7-*O*-neohesperidosyl **195** [43,94,355,356,357,358]
13	*Drynaria propinqua* (Wall. ex Mett.) Bedd	(-)-epiafzelechin 3-*O*-*β*-d-allopyranoside **13** [359]
14	*Drynaria quercifolia* (L.) J.Sm.	friedelin **196**, epifriedelinol **197**, *β*-amyrin **198**, *β*-sitosterol 11, 3-*β*-d-glucopyranoside **199**, 3,4-dihydroxybenzoic acid **200**, acetyllupeol **201** [97,360]
15	*Drynaria rigidula* (Sw.) Bedd.	fern-9(11)ene **202**, hop-22(29)-ene 156, *γ*-sitosterol **203**, 3,4-dihydroxybenzoic acid **200**, 4-hydroxybenzoic acid **204**, 4-hydroxyphenyl-1-(2-arabinopyranosyl)-tetrahydro-2*H*-pyran-3,4,5-triol **205**, 4-hydroxyphenyl-1-tetrahydro-2*H*-pyran-3,4,5-triol **206**, kaempferitrin **207**, 3,5-dihydroxy-flavone-7-*O*-*β*-rhamnopyranosyl-4′-*O*-*β*-glucopyranoside **208** [92,361]
16	*Phymatosorus scolopendria* (Burm. f.) Pic. Serm.	1,2-benzopyrone (coumarin) **209** [47]
17	*Pyrrosia lingua* (Thunb.) Farw.	diploptene **210**, *β*-sitosterol **11**, octanordammarane **211**, dammara-18(28),21-diene **212**, (18*S*)-18-hydroxydammar-21-en **213**, (18*R*)-18-hydroxydammar-21-ene **214**, (18*S*)-pyrrosialactone **215**, (18*R*)-pyrrosialactone **216**, (18*S*)-pyrrosialactol **217**, 3-deoxyocotillol **218**, dammara-18(28),21-diene **212**, cyclohopenol **219**, cyclohopanediol **220**, hop-22(29)-en-28-al **221** [362,363,364]
18	*Pyrrosia petiolosa* (Christ) Ching	*α*-tocopherol **222**, diploptene **210**, 24-methylene-9,19-cyclolanost-3*β*-yl acetate **223**, cycloeucalenol **224**, *β*-sitosterol 11, daucosterol 134, vanillic acid **225**, protocatechualdehyde **226**, hydrocaffeic acid **227**, caffeic acid **228**, 7-*O*-[6-*O*-(*α*-l-arabinofuranosyl)-*β*-D-glucopyranosyl]gossypetin **229**, kaempferol-3-*O*-*β*-d-glucopyranoside-7-*O*-*α*-l-arabinofuranoside **230** [365,366,367,368]
19	*Pyrrosia sheareri* (Baker) Ching	diploptene **210**, *β*-sitosterol 11, vanillic acid **225**, protocatechuic acid 189, mangiferin **73**, fumaric acid **231**, sucrose **232** [42]
	**Psilotaceae**	
20	*Psilotum nudum* (L.) P. Beauv	apigenin di-C-glycoside **233**, 7,4′,4′-tri-*O*-*β*-d-glucopyranoside **234**, 4′,4′-di-*O*-*β*-d-glucopyranoside **235**, 7,4′-di-*O*-*β*-d-glucopyranoside **236**, 3′-hydroxypsilotin (6-[4′-(*β*-D-glucopyranosyloxy)-3′-hydroxyphenyl]-5,6-dihydro-2-oxo-2*H*-pyran) **237**, 24-methylene-5*α*-lanost-8-en-3*β*-ol **238**, 24*β*-methyl-25-dehydrolophenol **239**, codisterol **240**, isofucosterol **241**, 24-methylene-25-hydroxyphenol **242**, avenasterol **243**, psilotin **244** [368,369,370,371]
	**Pteridaceae**	
21	*Acrostichum aureum* L.	quercetin 3-*O*-*β*-d-glucoside **245**, ponasterone A **246**, lupeol **247**, friedelin **196**, *β*-sitosterol **11**, stigmasterol **248**, campesterol **249**, tetracosanoic acid **250**, ursolic acid **251**, gallic acid **252**, (2*R*,3*S*)-sulfated pterosin C **253**, (2*S*,3*S*)-sulfated pterosin C **254**, (2*S*,3*S*)-pterosin C **255**, (2*R*)-pterosin P **256**, patriscabratine **257**, tetracosane **258**, quercetin-3-*O*-*β*-d-glucoside **259**, quercetin-3-*O*-*β*-d-glucosyl-(6→1)-*α*-l-rhamnoside **260**, quercetin-3-*O*-*α*-l-rhamnoside **261**, quercetin-3-*O*-*α*-l-rhamnosyl-7-*O*-*β*-d-glucoside **262**, kaempferol **153** [35,372,373,374]
22	*Selaginella involvens* (P.Beauv.) Spring	hexadecanoic acid **263**, stearic acid **264**, *β*-sitosterol **11**, stigmasterol **248**, amentoflavone **265**, *β*-d-glucopyranoside **266**, (3*β*)-cholest-5-en-3yl **267**, *β*-amyrin **198** [375]
	**Vittariaceae**	
23	*Vittaria elongate* Sw.	vittarin-A-F **268–273**, 3-*O*-acetylniduloic acid **274**, ethyl 3-*O*-acetylniduloate **275**, methyl 4-*O*-coumaroylquinate **276**, vittarilide-A, B **277**, **278**, vittariflavone **279**, methyl 4-*O*-caffeoylquinate **280**, ethyl 4-*O*-caffeoylquinate **281**, methyl 5-*O*-caffeoylquinate **282**, apigenin **132**, vitexin **283**, 5,7-dihydroxy-3′,4′,5′-trimethoxyflavone **284**, amentoflavone **265**, *trans*-*p*-coumaric acid **285**, methyl *trans*-*p*-coumarate **286**, methyl caffeate **287**, ferulic acid **288**, *p*-cresol **289**, 4-hydroxybenzaldehyde **290**, 4-hydroxybenzoic acid **204**, methyl 4-hydroxybenzoate **291**, protocatechualdehyde **226**, protocatechuic acid **189**, methyl protocatechuate **292**, vanillin **293**, vanillic acid **225** [119]
	**Non-Fern**	
	**Balsaminaceae**	
24	*Impatiens niamniamensis* Gilg (semi epiphytic)	*α*-*N*,*N*,*N*-trimethyltryptophan betaine **294** [129]
25	Convolvulaceace *(parasite)*	
26	*Cassytha filiformis* L.	*N*-(3,4-dimethoxyphenethyl)-4,5-methylenedioxy-2-nitrophenylacetamide **295**, actinodaphnine **296**, cassythine **297**, isoboldine **298**, cassameridine **299**, cassamedine **300**, lysicamine **301**, cathafiline **302**, cathaformine **303**, actinodaphnine **304**, *N*-methylactinodaphnine **305**, cathafiline **306**, cathaformine **307**, predicentrine **308**, ocoteine **309**, filiformine **310**, (+)-diasyringaresinol **311**, cathafiline **312**, cathaformine **313**, actinodaphnine **314**, *N*-methylactinodaphnine **315**, predicentrine **308**, ocoteine **316**, neolitsine **317**, dicentrine **318**, cassythine (cassyfiline) **319**, actinodaphnine **320**, 4-*O*-methylbalanophonin **321**, cassyformin **322**, isofiliformine **323**, cassythic acid **324**, cassythic acid **324**, cassythine **325**, neolitsine **326**, dicentrine **318**, 1,2-methylenedioxy-3,10,11-trimethoxyaporphine **327**, (-)-*O*-methylflavinatine **328**, (-)-salutaridine **329**, isohamnetin-3-*O*-*β*-glucoside **330**, isohamnetin-3-*O*-rutinoside **331** [134,354,376,377,378,379,380]
27	*Cuscuta australis* R.Br.	4-oic acid-7-oxo-kaurene-6*α*-*O*-*β*-d-glucoside **332**, thymidine **333**, caffeic acid **228**, *p*-coumaric acid **334**, caffeic-*β*-d-glucoside **335**, kaempferol **153**, quercetin **336**, astragalin **337**, hyperoside **338**, astragalin **339**, kaempferol **153**, quercetin **336**, *β*-sitosterol **11**, *β*-sitosterol 3-*O*-*β*-D-xylopyranoside **340** [381,382,383]
28	*Cuscuta reflexa* Roxb.	coumarin **341**, *α*-amyrin **342**, *β*-amyrin **198**, *α*-amyrin acetate **343**, *β*-amyrin acetate **344**, oleanolic acetate **345**, oleanolic acid **127**, stigmasterol **248**, lupeol **247**, stigmast-5-en-3-*O*-*β*-d-glucopyranoside tetraacetate **346**, stigmast-5-en-3-*O*-*β*-d-glucopyranoside **347**, stigmast-5-en-3-yl-acetate **348**, *β*-sitosterol 11, 3,5,7,3′-pentahydroxyflavanone (taxifolin) **349**, 3,5,7,4′-tetrahydroxyflavanone (aromadendrin) **350** [143,384,385]
	**Clusiaceae**	
29	*Clusia grandiflora* Splitg. (hemi epiphyte)	friedelin **196**, *β*-amyrin **198**, *β*-sitosterol 11, lupeol **247**, chamone I **351**, chamone II **352** [149,386]
	**Loganiaceae**	
30	*Fagraea auriculata* Jack. (semi epiphyte)	di-*O*-methylcrenatin **353**, potalioside B **354**, adoxosidic acid **355**, adoxoside **356**, (*þ*)-pinoresinol **357**, salicifoliol **358** [153]
	**Loranthaceae *(parasite)***	
31	*Dendrophthoe falcata* (L.f.)Ettingsh	3*β*-acetoxy-1*β*-(2-hydroxy-2-propoxy)-11*α*-hydroxy-olean-12-ene **359**, 3*β*-acetoxy-11*α*-ethoxy-1*β*-hydroxy-olean-12-ene **360**, 3*β*-acetoxy-1*β*-hydroxy-11*α*-methoxy-olean-12-ene **361**, 3*β*-acetoxy-1*β*,11*α*-dihydroxy-olean-12-ene **362**, 3*β*-acetoxy-1*β*,11*α*-dihydroxy-urs-12-ene **363**, 3*β*-acetoxy-urs-12-ene-11-one **364**, 3*β*-acetoxy-lup-20(29)-ene **365**, 30-nor-lup-3*β*-acetoxy-20-one **366**, (20*S*)-3*β*-acetoxy-lupan-29-oic acid **367**, kaempferol-3-*O*-*α*-l-rhamnopyranoside **368**, quercetin-3-*O*-*α*-l-rhamnopyranoside **369**, gallic acid **252** [387]
32	*Loranthus globosus* Roxb	(+)-catechin **168**, 3,4-dimethoxycinnamyl alcohol **370**, 3,4,5-trimethoxycinnamylalcohol **371** [163]
33	*Macrosolen cochinchinensis* (Lour.) Tiegh.	quercetin **336**, gallic acid **252**, orientin **372**, rutin **373**, quercetin-3-*O*-apiosyl(1→2)-[rhamnosyl(1→6)]-glucoside **374**, vicenin **375** [388]
34	*Scurrula atropurpurea* (Blume) Danser	octadeca-8,10,12-triynoic acid **376**, hexadec-8-ynoic acid **377**, hexadec-10-ynoic acid **378**, hexadeca-8,10-diynoic acid **379**, hexadeca-6,8,10-triynoic acid **380**, hexadeca-8,10,12-triynoic acid **381**, (*Z*)-9-octadecenoic acid **382**, (*Z*,*Z*)-octadeca-9,12-dienoic acid **383**, (*Z,Z,Z*)-octadeca-9,12,15-trienoicacid **384**, octadeca-8,10-diynoic acid **385**, (*Z*)-octadec-12-ene-8,10-diynoic acid **386**, octadeca-8,10,12-triynoic acid **376**, theobromine **387**, caffeine **388**, quercitrin **389**, rutin **373**, icariside B2 **390**, aviculin **391**, (+)-catechin **168**, (-)-epicatechin **144**, (-)-epicatechin-3-*O*-gallate **392**, (-)-epigallocatechin-3-*O*-gallate **393** [169,170]
35	*Scurrula ferruginea* (Jack) Danser	glycoside 4′-*O*-acetyl-quercitrin **394** [389]
36	*Scurrula parasitica* L.	(+)-catechin **168** [178]
	**Moraceae**	
37	*Ficus pumila* L.	(1*S*,4*S*,5*R*,6*R*,7*S*,10*S*)-1,4,6-trihydroxyeudesmane 6-*O*-*β*-d-glucopyranoside **39**, (1*S*,4*S*,5*S*,6*R*,7*R*,10*S*)-1,4-dihydroxymaaliane 1-*O*-*β*-d-glucopyranoside **396**, (23*Z*)-3*β*-acetoxycycloart-23-en-25-ol **39**, (23*Z*)-3*β*-acetoxyeupha-7,23-dien-25-ol **39**, (24RS)-3*β*-acetoxycycloart-25-en-24-ol **39**, (24*S*)-24-hydroxystigmast-4-en-3-one **400**, (24*S*)-stigmast-5-ene-3*β*,24-diol **401**, 10*α*,11-dihydroxycadin-4-ene 11-*O*-*β*-d-glucopyranoside **402**, 3*β*-acetoxy-(20*R*,22*E*,24*RS*)-20,24-dimethoxydammaran-22-en-25-ol **403**, 3*β*-acetoxy-(20*S*,22*E*,24*RS*)-20,24-dimethoxydammaran-22-en-25-ol **404**, 3*β*-acetoxy-20,21,22,23,24,25,26,27-octanordammaran-17*β*-ol **405**, 3*β*-acetoxy-22,23,24,25,26,27-hexanordammaran-20-one **406**, cycloartane-type triterpenoids **407**, triterpenoid **408** [390,391,392]
	**Orchidaceae**	
38	*Anoectochilus formosanus* Hayata	(6*R*,9*S*)-9-hydroxy-megastigma-4,7-dien-3-one-9-*O*-*β*-d-glucopyranoside **409**, (*R*)-(+)-3,4-dihydroxybutanoic acid *γ*-lactone **410**, 1-*O*-isopropyl-*β*-d-glucopyranoside **411**, 2-(*β*-d-glucopyranosyloxymethyl)-5-hydroxymethylfuran **412**, 3-(*R*)-3-*β*-d-glucopyranosyloxy-4-hydroxybutanoic acid **413**, 3-(*R*)-3-*β*-d-glucopyranosyloxybutanolide (kinsenoside) **414**, 4-(*β*-d-glucopyranosyloxy)benzyl alcohol **415**, corchoionoside C **416** [393]
39	*Anoectochilus roxburghii* (Blume)	24ξ-isopropenylcholesterol **417**, 5-hydroxy-3′,4′,7-trimethoxyflavonol-3-*O*-*β*-*D*-rutinoside **418**, 7-*O*-*β*-D-diglucoside **419**, 8-*C*-*β*-hydroxybenzylquercetin **420**, 8-*p-*hydroxybenzyl quercetin, **421**, anoectosterol **422**, campesterol **249**, cirsilineol **423**, daucosterol **134**, ferulic acid **288**, isorhamnetin **424**, isorhamnetin-3 **425**, isorhamnetin-3, 4′-*O*-*β*-d-diglucoside **426**, isorhamnetin-3-*O*-*β*-*D*-rutinoside **427**, isorhamnetin-7-*O*-*β*-d-glucopyranoside **428**, isorhamnetin-7-*O*-*β*-d-diglucoside **429**, kaempferol-3-*O*-*β*-d-glucopyranoside **430**, kaempferol-7-*O*-*β*-d-glucopyranoside **431**, *p*-coumaric acid **334**, *p*-hydroxybenzaldehyde **432**, quercetin **336**, quercetin 3′-O-*β*-d-glucopyranoside 433, quercetin 3-*O*-*β*-d-glucopyranoside **434**, quercetin 3-O-*β*-d-rutinoside **435**, quercetin 7-*O*-*β*-glucoside **436**, quercetin-7-*O*-*β*-*D*-[6′-*O*-(*trans*-feruloyl)]-glucopyranoside **437**, sitosterol **438**, stigmasterol **248**, succinic acid **439**, 3′,4′,7-trimethoxy-3,5-dihydroxyflavone **440**, 3-methoxyl-p-hydroxybenzaldehyde **441**, daucosterol **134**, daucosterol **134**, ferulic acid **288**, isorhamnetin-3-*O*-*β*-d-glucopyranoside **442**, isorhamnetin-3-*O*-*β*-D-rutinoside **443**, lanosterol **444**, methy1 4-*β*-d-glucopyranosyl-butanoate **445**, *o*-hydroxy phenol **446**, oleanolic acid **127**, palmitic acid **447**, *p*-hydroxy benzaldehyde **448**, *p*-hydroxy cinnamic acid **449**, *p*-hydroxybenzaldehyde **432**, rutin **373**, sorghumol 3-*O*-*E*-*p*-coumarate 450, sorghumol 3-*O*-*Z*-*p*-coumarate **451**, stearic acid **264**, succinic acid **452**, *β*-D-glucopyranosyl-(3*R*)-hydroxybutanolide **453**, *β*-sitosterol **11** [394,395,396,397,398,399,400,401,402]
40	*Bulbophyllum kwangtungense* Schltr.	10,11-dihydro-2,7-dimethoxy-3,4-methylenedioxydibenzo[*b,f*]oxepine **454**, 5-(2,3-dimethoxyphenethyl)-6-methylbenzo[*d*][1,3]dioxole **455**, 7,8-dihydro-3-hydroxy-12,13-methylenedioxy-11-methoxyldibenz[*b,f*]oxepin **456**, 7,8-dihydro-4-hydroxy-12,13-methylenedioxy-11-methoxyldibenz[b,f]oxepin **457**, 7,8-dihydro-5-hydroxy-12,13-methylenedioxy-11-methoxyldibenz [*b,f*]oxepin, **458**, cumulatin **459**, densiflorol A **460**, plicatol B **461** [219,403]
41	*Bulbophyllum odoratissimum* (Sm.) Lindl. ex Wall.	(+)-lyoniresinol-3a-*O*-*β*-d-glucopyranoside **462**, 3,5-dimethoxyphenethyl alcohol **463**, 3,7-dihydroxy-2,4,6-trimethoxyphenanthren **464**, 3-hydroxyphenethyl 4-*O*-(6′- O-*β*-apiofuranosyl)-*β*-d-glucopyranoside **465**, 3-methoxy-4-hydroxycinnamic aldehyde **466**, 3-methoxyphenethyl alc. 4-*O*-*β*-D-glucopynanoside **467**, 4-hydroxy-3,5-dimethoxybenzaldehyde **468**, 4-*O*-*β*-d-glucopynanoside **469**, 7-hydroxy-2,3,4-trimethoxy-9,10-dihydrophenanthrene **470**, batatasin III **471**, Bulbophyllanthrone **472**, bulbophythrins A, B **473**, **474**, Coelonin **475**, densiflorol B **476**, ethyl orsellinat **477**, gigantol **478**, moscatin **479**, *p*-hydroxyphenylpropionic acid **480**, *p*-hydroxyphenylpropionic methyl ester **481**, syringaldehyde **482**, syringin **483**, tristin **484**, vanillic acid **225** [223,224,404,405,406,407]
42	*Bulbophyllum vaginatum* (Lindl.) Rchb.f.	(±)-syringaresinol **485**, (2R*,3S*)-3-hydroxymethyl-9-methoxy-2-(4′-hydroxy-3′,5′-dimethoxyphenyl)-2,3,6,7-tetrahydrophenanthro [4,3-b]furan-5,11-diol **486**, 2,4-dimethoxyphenanthrene-3,7-diol **487**, 3,4,6-trimethenanthrene-2,7-diol **488**, 3,4,6-trimethoxy-9,10- dihydrophenanthrene-2,7-diol **489**, 3,4′,5-trihydroxy-3′-methoxybibenzyl (tristin) **490**, 3,4′-dihydroxy-5,5′-dimethoxybibenzyl **491**, 3,4-dihydroxybenzoic acid **200**, 3,4-dimethoxy-9,10- dihydrophenanthrene-2,7-diol (erianthridin) **492**, 3,4-dimethoxyphenanthrene-2,7-diol (nudol) **493**, 3,5-di- methoxy-9,10-dihydrophenanthrene-2,7-diol (6- methoxycoelonin) **494**, 3,5-dimeth- oxyphenanthrene-2,7-diol **495**, 3′-dihydroxy-5-methoxybibenzyl **496**, 4,4′,6,6′-tetramethoxy-[1,1′-biphenanthrene]-2,2′,3,3′,7,7′-hexol **497**, 4,6-dimethoxy-9,10-di- hydrophenanthrene-2,3,7-triol **498**, 4,6-dimethoxyphenanthrene-2,3,7-triol **499**, 4-methoxy-9,10- dihydrophenanthrene-2,7-diol (coelonin) **500**, 4-methoxyphenan- threne-2,7-diol (flavanthrinin) **501**, 4-methoxyphenanthrene- 2,3,5-triol (fimbriol B) **502**, 9,10- dihydrophenanthrenes **503**, dihydroferulic acid **504**, Friedelin **196**, *p*-coumaric acid, **334** [36,408,409]
43	*Catasetum barbatum* (Lindl.) Lindl.	2,7-dihydroxy-3,4,8-trimethoxyphenanthrene **505** [225]
44	*Cymbidium aloifolium* (L.) Sw.	aloifol I **506**, aloifol II **507**, 6-*O*-methylcoelonin **508**, batatasin III **471**, coelonin **475**, gigantol, **478**, 1-(4′-hydroxy-3′,5′-dimethoxyphenyl)-2-(3″-hydroxyphenyl)ethane **509**, 1-(4′-hydroxy-3′,5′-dimethoxyphenyl)-2-(4″-hydroxy-3″-methoxyphenyl)ethane **510**, 2,7-dihydroxy-4,6-dimethoxy-9,10-dihydrophenanthrene **511**, cymbinodin-A **512**, cymbinodin B **513** [410,411,412]
45	*Cymbidium goeringii* (Rchb.f.) Rchb.f.	*β*-sitosterol **11**, daucosterol **134**, ergosterol **514**, gigantol **478**, cymbidine A **515** [229,230,413]
46	*Dendrobium amoenum* Wall. ex Lindl.	amotin **516**, amoenin **517**, amoenumin **518**, amoenylin, isoamoenylin **519**, 3,4′-dihydroxy-5-methoxybibenzyl, **520**, 4,4′-dihydroxy-3,3′,5-trimethoxybibenzyl (moscatilin) **521** [414,415,416]
47	*Dendrobium chryseum* Rolfe	araxerol **522**, coumarin **341**, moscatilin **523**, chrysotobibenzyl **524**, chrysotoxin **525**, gigantol **478**, kaempferol **153**, *cis*-melilotoside **526**, defuscin **527**, dendroflorin **528**, dengibsin **529**, dihydromelilotoside **530**, naringenin **147**, *n*-octacosyl ferulate **531**, *trans*-melilotoside **532** [233,417]
48	*Dendrobium candidum* Wall. Ex Lindl.	(-)-loliolide **533**, (-)-secoisolariciresinol **534**, (-)syringaresinol **535**, (+)-lyoniresinol-3a-*O*-*β*-d-glucopyranoside **462**, (+)-syringaresinol-4-*β*-d-monoglucoside **536**, (1′*R*)-1′-(4-hydroxy-3,5-dimethoxylphenyl) propan-1′-ol 4-*O*-*β*-d-glucopyranoside **537**, (*E*)-*p*-Hydroxycinnamic acid **538**, 2,4,7-trihydroxy-9,10-dihydrophenanthrene **539**, 2-methoxyphenol-*O*-*β*-d-apiofuromosyl-(1→2)-*β*-d-glucopyranoside **540**, 3,4-dihydroxy-5,4′-dimethoxybibenzyl **541**, 3-*O*-methylgigantol **542**, 4,4′-dihydroxy-3,5-dimethoxybibenzyl **543**, 4′,5-dihydroxy-3,3′-dimethoxybibenzyl **544**, 4-allyl-2,6-dimethoxyphenylglucoside **545**, 4′-dihydroxy-5-methoxybibenzyl **546**, 5-hydroxymethyl-furaldehyde **547**, Adenosine **548**, Aduncin **549**, cis-feruloyl-*p*-hydroxybenzenethylamine **550**, coniferyl alcohol **551**, daucosterol **134**, defuscin **527**, denbinobin, **552**, dendrocandin A **553**, dendrocandin B **554**, dendrocandin C **555**, dendrocandin D **556**, dendrocandin E **557**, dendrocandins F—I **558–561**, dendromoniliside E **562**, dendrophenol **563**, dihydroresveratrol **564**, gigantol **478**, guanosine **565**, hentriacontane **8**, heptadecanoic acid **566**, hexadecanoic acid **263**, icariol A 2-4-*O*-*β*-d-glucopyranoside **567**, khaephuouside **568**, leonuriside A **569**, naringenin **147**, *n*-octacosyl ferulate **531**, *N*-trans-feruloyl tyramine **570**, *n*-triacontyl *cis*-*p*-coumarate **571**, *p*-hydroxy-phenylpropionic acid **480**, sucrose **232**, syringaresinol **572**, syringaresinol-4,4′-*O*-bis-*β*-d-glucoside **573**, *trans*-cinnamoyl-*p*-hydroxybenzenethylamine **574**, uridine **575**, vanillyl alcohol **576**, *β*-sitosterol **11** [237,238,239,418,419,420]
49	*Dendrobium chrysanthum* Wall. ex Lindl.	(2*S*)-*N*-*cis*-cinnamoyl-2-oxopropyrrolidine **577**, (2*S*)-*N*-*trans*-cinnamoyl-2-oxopropyrrolidine **578**, (*þ*)-lyoniresinol **579**, 2,5-dihydroxy-4,9-dimethoxylphenanthrene **580**, 4,4′-dihydroxy-3,3′,5-trimethoxybibenzyl **581**, 7,70-bis-(4-hydroxy-3,5-dimethoxyphenyl)-8,80-dihydroxymethyl-tetrahydrofuran-4-*β*-d-glucoside **582**, chrysophanol **583**, chrysotobibenzyl **524**, chrysotobibenzyl **524**, chrysotoxin **525**, crepidatin **584**, crepidatin **584**, dehydrodiconiferyl alcohol-4-*β*-d-glucoside **585**, denchrysans A, B **586**, **587**, denchryside A **588**, denchryside B **589**, dendrochrysanene **590**, dendroflorin **528**, dengibsin **529**, dengibsin **529**, emodin **591**, gigantol **478**, moscatilin **523**, moscatilin **523**, moscatin **479**, physcion **592**, *β*-sitosterol **11** [226,417,421,422,423,424]
50	*Dendrobium fimbriatum* Hook.	2-hydroxyethyl caffeate **593**, ayapin **594**, chrysophanol **583**, chrysotobibenzyl (I) **595**, confusarin **596**, crepidatin **584**, defuscin **527**, denhydroshizukanolide **597**, fimbriatone **598**, *n*-dotriacontanoic acid **599**, *n*-octacosyl ferulate **531**, *n*-triacontyl *cis*-*p*-coumarate **571**, physcion **592**, rhein **600**, scopolin methyl ether **601**, *β*-sitosterol **11** [425,426]
51	*Dendrobium loddigesii* Rolfe	dendrophenol (4,4′-dihydroxy-3,3′,5-trimethoxybibenzyl) **563**, loddigesiinols A-D **602-605**, moscatilin **523**, moscatilin diacetate **606**, moscatin **479**, shihunidine **607**, shihunine **608**, stilbenes **609** [250,251,252]
52	*Dendrobium moniliforme* (L.) Sw.	heptacosane **610**, 3,4-dihydroxy-4′,5-dimethoxy bibenzyl **611**, 3,4-dihydroxy-5,4′-dimethoxy bibenzyl **612**, 4-methoxybenzaldehyde **613**, a known alkaloid 6-hydroxynobiline **614**, alkyl 4′-hydroxy-*cis*-cinnamates **615**, alkyl ferulates **616**, daucosterol **134**, denbinobin **552**, denbinobin, alkyl 4′-hydroxy-trans-cinnamates **617**, dendromoniliside E **562**, ethyl linolenates **618**, heptatriaconsanoic acid **619**, linoleic acid **620**, methyl linolenates **621**, moniliformin **622**, moniline **623**, *n*-nonacosane **624**, *n*-octacosyl ferulate **531**, *n*-triacontyl *p*-hydroxy-*cis*-cinnamate **625**, octacosanyl hexadecanoate **626**, phytosterols **627**, stigmast-4-en-3-one **628**, vanillin **293**, *α*-dihydropicrotoxinin **629**, *β*-sitosterol **11** [255,427,428,429,430,431]
53	*Dendrobium moschatum* (Buch.-Ham) S.w	moscatin **479**, moscatilin **523** [432,433]
54	*Dendrobium nobile* Lindl.	10,12-dihydroxypicrotoxane **630**, 10*β*,13,14-trihydroxyalloaromadendrane **631**, 3,4,8-trimethoxyphenanthrene-2,5-diol **632**, 3,4′-dihydroxy-5,5′-dimethoxydihydrostilbene **633**, 3-*O*-methylgigantol **542**, 5,7-dimethoxyphenanthrene-2,6-diol **634**, 6-hydroxy-dendrobine (dendramine) **635**, 6-hydroxy-dendroxine **636**, 6*α*,10,12-trihydroxypicrotoxane **637**, 7,12-dihydroxy-5-hydroxymethyl-11-isopropyl-6-methyl-9-oxatricyclo [6.2.1.0^2,6^]undecan-10-one-15-*O*-*β*-d-glucopyranoside **638**, batatasin III **471**, bullatantirol **639**, chrysotobibenzyl **524**, coelonin **475**, crepidatin **584**, denbinobin **552**, dendrobane A **640**, dendrobin A,7 chrysotoxine **641**, dendrobine **642**, dendrobiumane **643**, dendrodensiflorol, **644**, dendroflorin **528**, dendronobilin A-I **645–653**, dendronobilin J **654**, dendronobiline A **655**, dendronobilosides A, B **656**, **657**, dendronophenol A-B **658**, **659**, dendroside A **660**, dendroside E-G **661–663**, dendroxineo **664**, ephemeranthol A **665**, epheneranthol C **666**, erianthridin **667**, fimbriol-B **668**, flavanthridin **669**, gigantol **478**, hircinol **670**, lusianthridin **671**, moscatilin **523**, moscatilin **523**, moscatin, **479**, gigantol **478**, nobilin D-E **672**, **673**, nobilone **674**, nobilonine **675**, stigmasterol **248**, *β*-sitosterol **11**, *β*-sitosterol glucoside **12** [38,261,262,263,264,267,433,434,435,436,437,438]
55	*Epidendrum strobiliferum* Rchb.f.	24-methylenecycloartanol **676**, campesterol **249**, pholidotin **677**, stigmasterol **248**, *β*-sitosterol **11** [272]
56	*Epidendrum rigidum* Jacq.	2,3-dimethoxy-9,10-dihydrophenathrene-4,7-diol **678**, 24-methyl-9,19-cyclolanostane-25-en-3*β*-ol **679**, 3,4,9-trimethoxyphenanthrene-2,5-diol **680**, apigenin **132**, batatasin III **471**, gigantol **478**, isovitexin **681**, stilbenoids I-IV **682–685**, triterterpenoids 24,24-dimethyl-9,19-cyclolanostane-25-en-3*β*-ol **686**, vitexin **283** [274]
57	*Mycaranthes pannea* (Lindl.) S.C.Chen & J.J.Wood	Acervatol **687**, acervatone **688**, flavanthridin **669**, flavanthrinin **689** [276]
58	*Camaridium densum* (Lindl.) M.A.Blanco	2,5-dihydroxy-3,4-dimethoxyphenanthrene **690**, 2,5-dihydroxy-3,4-dimethoxyphenanthrene **690**, 9,10-dihydro-2,5-dihydroxy-3,4-dimethoxyphenanthrene **691**, 9,10-dihydro-2,7-dihydroxy-3,4-dimethoxyphenanthrene **692**, erianthridin **667**, fimbriol-A **693**, gymnopusin **694**, nudol **695** [37,439]
59	*Nidema boothii* (Lindl.) Schltr.	1,5,7-trimethoxy-9,10-dihydrophenanthrene-2,6-diol, **696**, 1,5,7-trimethoxyphenanthrene-2,6-diol **697**, 2,4-dimethoxyphenanthrene-3,7-diol **488**, 9,19-cyclolanosta-24,24-dimethyl-25-en-3*β*-yl trans-*p*-hydroxycinnamate **698**, aloifol II 507, batatasin III 471, ephemeranthol B **699**, ephemeranthoquinone **700**, gigantol **478**, lusianthridin **671**, nidemin **701**, nidemone **702** [282,440]
60	*Pholidota articulata* Lindl.	2,7-dihydroxy-3,4,6-trimethoxyla 9, 10-dihydrophenanthrene flavidin **703**, 2,7-dihydroxyll-methoxy-9,10-dihydrophenanthrene (coelonin) **704**, 9, 10-dihydrophenanthrenes **705**, coelogin **706**, coeloginin **707**, flavidin **708**, flavidinin **709**, oxoflavidinin **710** [441]
61	*Pholidota chinensis* Lindl.	(*E*)-2′,3,3′-trihydroxy-5-methoxystilbene (pholidotol C) **711**, (*Z*)-3,3′-hydroxy-5-methoxystilbene (pholidotol D) **712**, 2,4,7-trihydroxy-9,10-dihydrophenanthrene **539**, 2,5-dimethoxy-3,4,3′,4′-bis(dimethylenedioxy)bibenzyl **713**, 3,4′-dihydroxy-3′,5-dimethoxybibenzyl **714**, 3,4-dihydroxy-4-methoxydihydrostilbene **715**, 4,4′-dihydroxydiphenylmethane **716**, 4,5-dihydroxy-2-methoxy-9,10-dihydrophenanthrene **717**, 5,3′-dihydroxy-2,3-(methylenedioxy)bibenzyl **718**, 9,10-dihydro-2,4-dihydroxy-7-methoxyphenanthrene **719**, batatasin III **471**, blestrianol A **720**, blestrin A **721**, bulbophylol B **722**, cannabidihydrophenanthrene **723**, coelonin **475**, coelonin **475**, cyclopholidone **724**, cyclopholidone **724**, cyclopholidonol **725**, cyclopholidonol **725**, erianthridin **667**, eulophiol **726**, flavanthrin **727**, flavanthrin **727**, gymconpin C **728**, hircinol **670**, lusianthridin **671**, lusianthridin, **671**, phochinenins A – F **729–734**, phochinenins G-L **735–740**, pholidotols A-B **741**, **742**, 3,4-dihydroxy-5-methoxydihydrostilbene **743**, phoyunnanin D **744**, *p*-hydroxybenzaldehyde **432**, *p*-hydroxybenzyl alcohol **745**, protocatechuic aldehyde **746**, resveratrol **747**, thunalbene **748**, thunalbene **749**, *trans*-3-3-dihydroxy-2,5-dimthoxystilbene **750**, *trans*-3-hydroxy-2,3,5-trimthoxystilbene **751**, *β*-daucosterol **752** [285,286,442,443,444,445]
62	*Scaphyglottis livida* (Lindl.) Schltr.	24,24,dimethyl-9,19-cyclolanosta-9(11),25-dien-3-one (cyclobalanone) **753**, 3,4′-dihydroxy-3′,4,5-trimetoxybibenzyl **754**, 3,4′-dihydroxy-3′,5-dimethoxybibenzyl **714**, 3,7-dihydroxy-2,4,8-trimethoxyphenanthrene **755**, 3,7-dihydroxy-2,4-dimethoxyphenanthrene **756**, 5*α*-lanosta-24,24-dimethyl-9(11),25-dien-3*β*-ol **757**, batatasin III **471**, coelonin **475**, gigantol **478**, nidemin **701** [287,288,440]
63	*Papilionanthe teres* (Roxb.) Schltr.	eucomic acid **758**, vandaterosides I-III **759–761** [295]
64	*Vanda tessellate* (Roxb.) Hook. ex G. Don.	Oxotessallatin **762** [446]
	**Piperaceae**	
65	*Peperomia galioides* Kunth	(+)-epi-*α*-bisabolol **763**, galopiperone **764**, grifolic acid **765**, grifolin **766**, hydropiperone **767**, piperogalin **768**, piperogalone **769** [447,448,449]
66	*Piper retrofractum* Vahl	28-methylnonacos-27-en-1-oic acid **770**, 3-methyl-5-decanoylpyridine **771**, caffeic acid **228**, di-methyl 3,4-bis(4-hydroxyphenyl)-1,2-cyclobutanedicarboxylate **772**, esculetin **773**, methyl piperate **774**, *N*-isobutyleicosa-2,4-dienamide **775**, *p*-coumaric acid **334**, pipereicosalidine **776**, piperine **777**, piperine **777**, pipernonaline **778**, piperoctadecalidine **779**, retrofractamide-D **780**, retrofractamides A, C **781**, **782**, uracil **783**, uridine **575**, vitexin **283**, vitexin 2′-*O*-*β*-glucopyranoside **784**, *β*-d-glucopyranoside **266**, *β*-sitosterol **11** [301,306,450,451,452,453]
	**Rubiaceae**	
67	*Hydnophytum formicarum* Jack	4-aminophenyl acetate **785**, 7,3′,5′-trihydroxyflavone **786**, butein **787**, butin **788**, Isoliquiritigenin **789**, protocatechualdehyde **226**, stigmast-4-en-3-one **628**, stigmasterol **248**, *β*-sitosterol **11** [313,361]
	**Viscaceae**	
68	*Viscum articulatum* Burm.f.	(2*S*)-5,3,4-trihydroxyflavanone 7-*O*-*β*-d-glucoside **790**, (2*S*)-homoeriodictyol **791**, (2*S*)-homoeriodictyol 7-*O*-*β*-d-glucoside **792**, (2*S*)-naringenin 7-*O*-*β*-d-glucoside **793**, (2*S*)-pinocembrin 7-*O*-[cinnamoyl(1→5)-*β*-d-apiosyl(1→2)]-*β*-d-glucoside **794**, (2*S*)-pinocembrin 7-*O*-[*β*-d-apiosyl(1→2)]-*β*-d-glucoside (1) **795**, (2*S*)-pinocembrin 7-*O*-*β*-d-glucoside **796**, (4′-hydroxy-2′,3′,6′,3′′-tetramethoxy-1,3-diphenylpropane)-4′′-*O*-*β*-d-glucopyranoside **797**, 1-*O*-benzyl-[5-*O*-benzoyl-*β*-Dapiofuranosyl(1→2)]-*β*-d-glucopyranoside **798**, 2-deoxy-*epi*-inositol **799**, 2-phenylethanol **800**, 4-*β*-d-glucosyloxy-3-hydroxy-benzoic acid **801**, 4′-hydroxy-7,3′-dimethoxyflavan-5-*O*-*β*-d-glucopyranoside **802**, 4-*O*-cinnamoyl quinic acid **803**, 5,3′,4′-trihydroxyflavanone-7-*O*-*β*-d-glucopyranoside **804**, 5,4′-dihydroxyflavanone-7-*O*-*β*-d-lucopyranoside **805**, 7-*O*-*β*-d-glucopyranoside **806**, botulin **807**, betulin **808**, betulinic acid **809**, cinnamic acid methyl ester **810**, diphenylpropane glycoside **811**, eriodictyol 7-*O*-*β*-d-glucopyranoside **812**, homoeriodictyol 7-*O*-*β*-d-glucopyranoside **813**, homoeriodictyol-7-*O*-*β*-d-glucopyranoside **814**, homoeriodictyol-7-*O*-*β*-d-glucopyranoside-4′-*O*-*β*-d-(5′′′-cinnamoyl)apiofuranoside **815**, homoeriodictyol-7-*O*-*β*-d-glucopyranoside-4′-*O*-*β*-d-apiofuranoside **816**, lupenyl acetate **817**, lupeol **247**, lupeol acetate **818**, lupeol palmitate **819**, lupeol stearate **820**, lycorin **821**, methylparaben **822**, naringenin 7-*O*-*β*-d-glucopyranoside **823**, Oleanolic acid **127**, *p*-hydroxybenzaldehyde **432**, *p*-hydroxy-benzoic acid **824**, pinocembrin **825**, pinocembrin 7-*O*-*β*-d-glucopyranoside **826**, pinocembrin-7-*O*-[cinnamoyl (1→5)-*β*-d-apiofuranosyl (1→2)]-*β*-d-glucopyranoside **827**, pinocembrin-7-*O*-*β*-d-apio furanosyl(1→2)-*β*-d-glucopyranoside **828**, pinocembrin-7-*O*-*β*-d-apiofuranosyl-(1→5)-*β*-d-apiofuranosyl-(1→2)-*β*-d-glucopyranoside **829**, protocatechuic acid 189, vanillin **293**, visartisides A-C **830**, **831**, **832**, visartisides D-F (4–6) **833**, **834**, **835**, viscumitol **836**, *α*-amyrin **342**, *β*-amyrin acetate **837**, *β*-sitosterol **11** [319,320,321,322,323,454,455,456]
69	*Viscum ovalifolium* DC	3-*O*-*α*-l-arabinopyranoyl-hederagenin-28-*O*-*β*-d-glucopyranosyl-(1→6)-*β*-d-glucopyranoside **838**, gypsogenic acid **839**, hederagenin **840**, hederagenin-3-*O*-*α*-l-arabinopyranoside **841**, hederagenin-3-*O*-*α*-l-arabinopyranoyl-(2→1)-*O*-*β*-d-glucopyranoside **842**, lupeol acetate **818**, lupeol palmitate **819**, oleanolic acid **127**, lupeol stearate **820**, *β*-amyrin **198**, *β*-amyrin acetate **344** [457,458]
